# CRB3 and NF2 orchestrate cytoskeletal dynamics to control epithelial barrier assembly

**DOI:** 10.1172/jci.insight.196350

**Published:** 2025-10-22

**Authors:** Shuling Fan, Saranyaraajan Varadarajan, Vicky Garcia-Hernandez, Sven Flemming, Arturo Raya-Sandino, Ben Margolis, Charles A. Parkos, Asma Nusrat

**Affiliations:** 1 Department of Pathology, and; 2 Department of Internal Medicine, University of Michigan Medical School, Ann Arbor, Michigan, USA.

**Keywords:** Cell biology, Gastroenterology, Inflammation, Cell migration/adhesion, Cytokines, Tight junctions

## Abstract

The gastrointestinal epithelium depends on the apical junctional complex (AJC), composed of tight and adherens junctions, to regulate barrier function. Here, we identify the apical polarity protein Crumbs homolog 3 (CRB3) as an important regulator of AJC assembly and barrier function in intestinal epithelium. Using primary murine colonic epithelial cells (colonoids) from inducible, conditional *Crb3*-knockout (*Crb3*^ER*Δ*IEC^) and control (*Crb3^fl/fl^*) mice, we show that CRB3 deficiency compromised barrier function that was associated with a hypercontractile perijunctional actomyosin network and impaired AJC assembly. Loss of CRB3 exacerbated proinflammatory cytokine–induced AJC remodeling, leading to increased intestinal permeability. *Crb3*^ER*Δ*IEC^ cells exhibited increased RhoA activity and junctional tension, which could be reversed by ROCK-II or myosin II inhibition, restoring junctional architecture. Mechanistically, CRB3A interacts with the actin cytoskeletal linker protein, Merlin (NF2) via its FERM-binding domain, and NF2 knockdown phenocopied CRB3 loss, suggesting their cooperative role in AJC assembly. These findings establish CRB3 and NF2 signaling as key regulators of perijunctional actomyosin contractility and AJC organization during both de novo junctional assembly and inflammation-induced remodeling. This work defines a CRB3- and NF2-dependent pathway by which epithelial cells regulate mechanical tension to coordinate barrier assembly during homeostasis and junctional remodeling under inflammatory stress.

## Abstract 2

CRB3–NF2 mediated RhoA/ROCK signaling is key regulator of perijunctional actomyosin contractility and intestinal epithelial barrier function during both de novo junction assembly and inflammation induced remodeling.

## Introduction

In the gut, a monolayer of epithelial cells forms a selective barrier that protects underlying tissues from antigens and pathogens while selectively regulating the movement of nutrients, ions, and water. This critical epithelial barrier function is controlled by intercellular junctions, including apical tight junctions (TJs) and adherens junctions (AJs), which are collectively referred to as the apical junctional complex (AJC). The assembly and maintenance of proteins within the AJC are regulated by signaling proteins, mechanical forces, and the interaction of AJC proteins with the underlying perijunctional actomyosin cytoskeleton (1–3). Thus, failure to assemble or maintain the AJC results in a compromised epithelial barrier function, which has been implicated in the pathogenesis of chronic inflammatory mucosal diseases, such as inflammatory bowel disease (IBD) (4–6). Given the critical role of the AJC in controlling epithelial barrier function, it is important to understand the mechanisms that govern its formation and maintenance in organ systems like the intestine.

The Crumbs (CRB) family of proteins plays an important role in maintaining apical-basal polarity and regulating intercellular junctions (7–12), organization of perijunctional actomyosin cytoskeleton (13–15), cell proliferation (16–20), and morphogenesis in epithelial tissues (21, 22). Three Crumbs paralogs (CRB1, CRB2, and CRB3) are expressed in different tissues. CRB1 is widely expressed in the eye (23); CRB2 is found in the brain, kidney, lung, and heart (24); and CRB3 is expressed in various epithelial tissues, including the lungs, kidney, and intestine (22). In mammals, CRB3 exists as 2 isoforms, CRB3A and CRB3B, which share identical extracellular and transmembrane domains but differ in their intracellular C-terminal regions (25, 26). Several in vitro studies have revealed that loss of CRB3 leads to defects in cell polarity and impaired epithelial junctions, contributing to tumor progression and metastasis in epithelial tissues (27, 28).

The intracellular C-terminal region of CRB3 contains a juxtamembrane FERM (Band 4.1/Ezrin/Radixin/Moesin) binding domain (FBD) and a C-terminal PDZ (PSD-95/Dlg/ZO-1) binding motif (PBM). The PBM of CRB3 interacts with other polarity complex proteins, including protein associated with Lin seven (PALS) and PALS1-associated TJ protein (PATJ), PAR6, and aPKC via the PALS-PATJ-PAR-aPKC complex (9, 29, 30). CRB3’s interaction with the PALS-PATJ-PAR-aPKC complex has a well-established role in determining apical-basal polarity in epithelial cells (12, 31). The FBD of CRB3 interacts with FBD-containing proteins that connect the apical plasma membrane to the underlying actin cytoskeleton (7). While previous studies using transformed epithelial cell lines have suggested a potential role for CRB3 in regulating barrier function (7, 26), its specific role and underlying mechanisms in AJC regulation during junctional remodeling in primary mammalian intestinal epithelial cells (IECs) remain undefined. Specifically, the contribution of the CRB3 FBD to the regulation of intercellular junctions and epithelial barrier function has yet to be explored.

In this study, we investigated how CRB3A, hereafter referred to as CRB3, regulates AJC assembly and therefore barrier function in primary murine IECs. We generated mice with an inducible IEC-specific knockout (KO) of CRB3 (*Crb3^ERΔIEC^*). These mice were used to establish primary colonic epithelial cultures, referred to as colonoids. KO of CRB3 in colonoids led to compromised epithelial barrier function during de novo junctional assembly and after proinflammatory cytokine exposure. Furthermore, increased mucosal permeability was identified after proinflammatory cytokine treatment of *Crb3^ERΔIEC^* mice compared with *Crb3^fl/fl^* control mice. Our in vivo analysis was complemented with a marked reduction in ZO-1 intensity and mislocalization of E-cadherin within the AJC of proinflammatory cytokine–treated *Crb3^ERΔIEC^* colonoids compared with *Crb3^fl/fl^* controls. Furthermore, the compromised epithelial barrier was associated with disorganization of the perijunctional actomyosin belt. These changes were associated with increased spatial activation of RhoA at the AJC and the formation of a hypercontractile perijunctional actomyosin ring, mediated by RhoA/ROCK-II signaling. We identified that CRB3A interacts with the Band 4.1 family of actin cytoskeletal linker protein, Merlin (NF2), via its FBD. Interestingly, the knockdown (KD) of NF2 in cultured IECs phenocopied the effect of CRB3 loss on perijunctional actomyosin architecture and barrier integrity. Using *Crb3^ERΔIEC^* colonoids, we demonstrated that reintroducing the full-length CRB3A restores appropriate localization of AJC proteins and organization of perijunctional actomyosin. However, CRB3A mutants lacking either the PBM or FBD failed to rescue AJC assembly, highlighting the essential role of these domains in CRB3-mediated regulation of AJC assembly. In summary, our findings highlight important roles for CRB3 and NF2 in coordinating perijunctional actomyosin organization and promoting AJC assembly and barrier function in IECs.

## Results

### Expression of CRB3 in the AJC of IECs and generation of Villin-Cre-ER^T2^ inducible Crb3-KO (Crb3^ERΔIEC^) mice.

**Figure 1:**
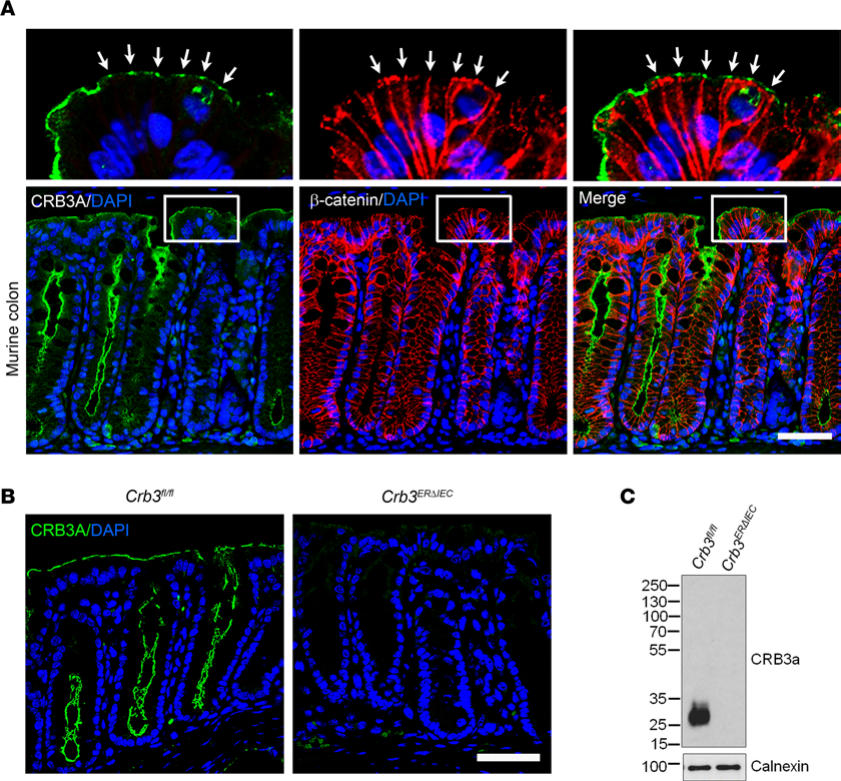
Inducible intestinal epithelial knockout of CRB3 (*Crb3^ERΔIEC^*). (**A**) Confocal image of murine colonic tissue sections immunostained for CRB3A (green), β-catenin (red), and nuclei (DAPI, blue). White rectangles represent the zoomed-in areas shown above the images. White arrows indicate tight junctions of colonic epithelial cells. Scale bar: 50 μm. (**B**) Immunofluorescent labeling of colonic tissue sections of tamoxifen-treated *Crb3^fl/fl^* and *Crb3*^ER*Δ*IEC^ mice showing CRB3A (green) and nuclei (DAPI, blue). Scale bar: 50 μm. (**C**) Immunoblotting of CRB3A and calnexin (loading control) in colonoids harvested from tamoxifen-treated *Crb3^fl/fl^* and *Crb3*^ER*Δ*IEC^ mice. Molecular weight markers are displayed on the left. *n* = 3 independent experiments, 2 technical replicates each.

To explore the spatial localization of intestinal epithelial CRB3 in the crypt-luminal axis, we first immunolocalized CRB3 in murine colonic tissue sections. As shown in Figure 1A, CRB3 localizes in the apical membrane and at the TJ (Figure 1A, white arrows), in contrast with β-catenin that spans the lateral membrane in murine colonic epithelial cells. An analogous CRB3 distribution was identified in healthy human colonic tissue sections (Supplemental Figure 1A; supplemental material available online with this article; https://doi.org/10.1172/jci.insight.196350DS1). Additionally, an analogous distribution of CRB3 in TJs of colonic epithelial cells was observed with polarity proteins PALS1, PATJ, and PAR3 (Supplemental Figure 1D, first column).

Considering the pivotal role of CRB3 in regulating cell polarity and epithelial morphogenesis (22), we examined the effects of CRB3 loss on colonic crypt architecture. Previous studies have reported that germline deletion of CRB3 leads to neonatal lethality in mice (22). Therefore, we generated mice with tamoxifen-inducible deletion of *Crb3* specifically in IECs (*Crb3^ERΔIEC^*) by crossing *Crb3^fl/fl^* mice with mice expressing an inducible mutated estrogen receptor fused to Cre recombinase under the control of the Villin promoter (Villin-Cre-ER^T2^) (21). Cre-ER^T2^–mediated recombination efficiently excised exon 3, which expresses both splice isoforms of Crb3, Crb3a and Crb3b, in *Crb3^ERΔIEC^* mice (21). The efficiency of CRB3 KO was confirmed by immunofluorescent labeling of the murine colonic mucosa (Figure 1B). Additionally, CRB3 deletion was confirmed by immunoblotting, using cells harvested from *Crb3^ERΔIEC^* mice and comparing them to *Crb3^fl/fl^* matched littermates (Figure 1C). Immunoblotting using an antibody that recognizes all 3 isoforms of Crumbs (CRB1, CRB2, and CRB3) further showed that CRB3 is the only Crumbs protein expressed in murine IECs, with a molecular weight of approximately 25 kDa (21) (Figure 1C and Supplemental Figure 1B, red arrow). Notably, the colonic mucosa of the *Crb3^ERΔIEC^* mice revealed no gross changes in mucosal architecture and was indistinguishable from that of littermate *Crb3^fl/fl^* mice, as shown by immunofluorescent labeling/confocal microscopy and histology of the colon (Figure 1B and Supplemental Figure 1, C and D). Additionally, immunostaining of colons derived from *Crb3^fl/fl^* and *Crb3^ERΔIEC^* mice revealed decreased junctional localization of CRB3 binding partner proteins (PALS1 and PATJ) in *Crb3^ERΔIEC^* mice, while PAR3 remained unchanged (Supplemental Figure 1D). Collectively, our results demonstrate efficient CRB3 KO in *Crb3^ERΔIEC^* IECs in a viable in vivo mouse model without a substantial change in the architecture of the colonic crypts.

### Intestinal epithelial CRB3 regulates localization of AJC proteins, organization of perijunctional actin cytoskeleton, and barrier function during junctional assembly and remodeling.

**Figure 2:**
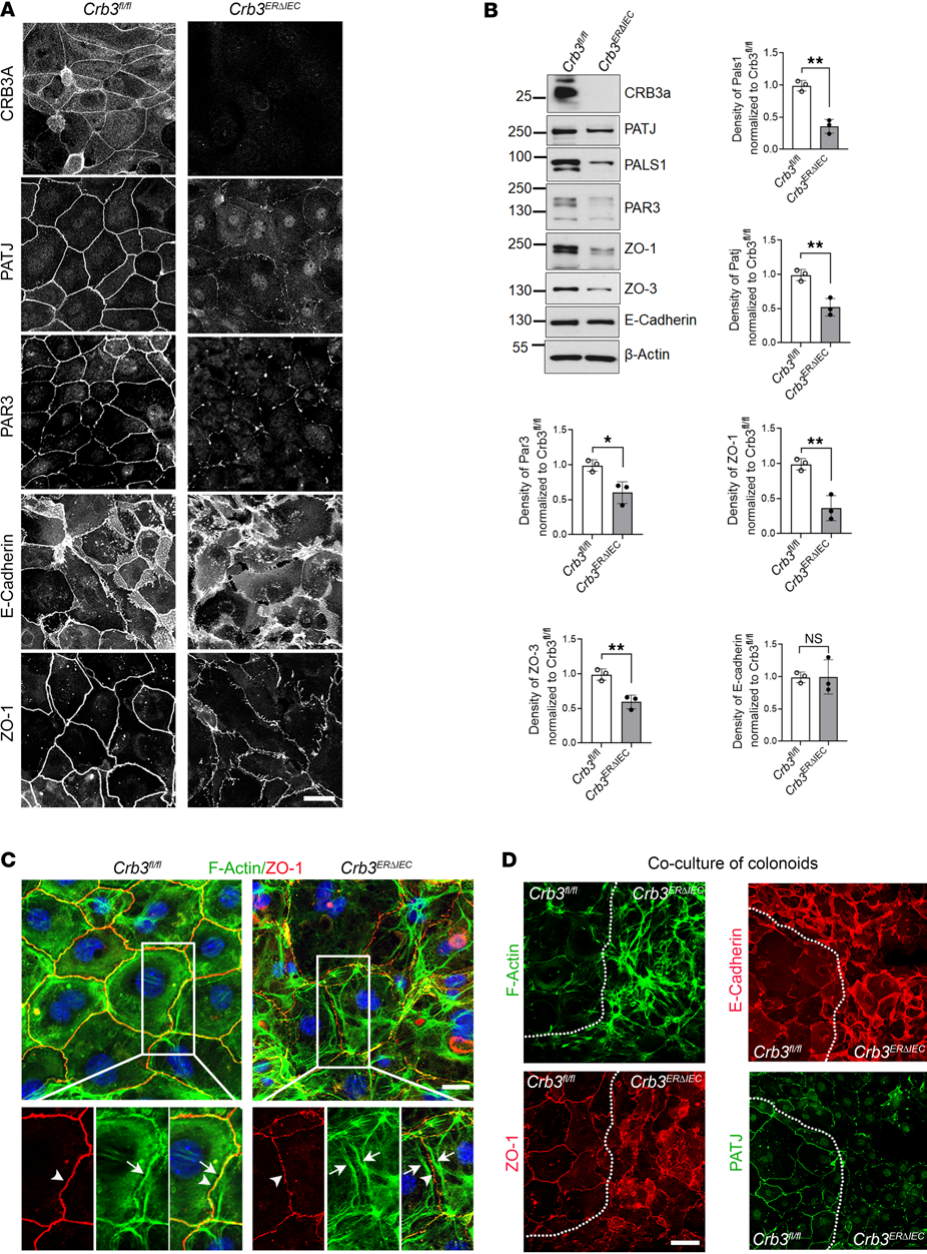
CRB3 regulates junctional localization of AJC proteins and organization of perijunctional actin cytoskeleton in IECs. (**A**) Representative confocal images of colonoids derived from *Crb3^fl/fl^* and *Crb3*^ER*Δ*IEC^ mice during junctional assembly (subconfluent) immunostained for polarity proteins (CRB3, PATJ, PAR3), AJ protein (E-cadherin), and TJ protein (ZO-1) Scale bar: 25 μm. *n* = 3 independent experiments, 3 technical replicates each. (**B**) Immunoblotting of colonoids derived from *Crb3^fl/fl^* and *Crb3*^ER*Δ*IEC^ mice during junctional assembly (subconfluent) for polarity proteins (CRB3, PATJ, PALS1, PAR3), AJ protein (E-cadherin), TJ protein (ZO-1, ZO-3), and loading control (β-actin). Graph showing the quantification of immunoblots in **B**. Data are mean ± SD, *n* = 3 experiments. *P ≤ 0.05, **P ≤ 0.01 by 2-tailed Student’s *t* test. (**C**) Representative confocal images of F-actin (phalloidin, green), TJ protein (ZO-1, red), and nuclei (DAPI, blue) in colonoids derived from *Crb3^fl/fl^* and *Crb3*^ER*Δ*IEC^ mice. White rectangles represent the zoomed-in areas shown below the composite images; arrowheads mark ZO-1; single arrow marks junctional localization of F-actin; double arrow in *Crb3*^ER*Δ*IEC^ marks railroad F-actin localization. Scale bar: 25 μm. *n* = 3 independent experiments, 3 technical replicates each. (**D**) Coculture of colonoids derived from *Crb3^fl/fl^* and *Crb3*^ER*Δ*IEC^ mice stained for F-actin (phalloidin, green), ZO-1 (red), PATJ (green), and E-cadherin (red). White dotted line marks the boundary between cells expressing CRB3 and cells lacking CRB3. Scale bar: 25 μm. *n* = 3 independent experiments, 3 technical replicates each.

The interaction of CRB3 with PALS1-PATJ and PAR3-PAR6-aPKC protein complexes in epithelial cells is important for regulating the AJC, which includes the TJ and AJ. Immunostaining and confocal imaging of subconfluent colonoids with assembling intercellular junctions harvested from *Crb3^fl/fl^* and *Crb3^ERΔIEC^* mice revealed discontinuous junctional localization of PATJ and PAR3 in the absence of CRB3 (Figure 2A). This finding corroborates previous reports, demonstrating that CRB3 stabilizes its binding partners PATJ and PALS1 at cell-cell junctions. Additionally, PAR3 and the TJ scaffold protein, ZO-1, exhibited discontinuous labeling and reduced intensity at TJs of colonoids in the absence of CRB3 (Figure 2, A and C). E-cadherin was visualized as linear staining in the junction plasma membrane of epithelial cells derived from *Crb3^fl/fl^* mice. However, in IECs lacking CRB3 (*Crb3^ERΔIEC^*), E-cadherin was visualized as diffuse and disrupted staining in the lateral plasma membrane (Figure 2A). Additionally, immunoblotting of colonoids derived from *Crb3^fl/fl^* and *Crb3^ERΔIEC^* mice revealed decreased CRB3 binding partner proteins PATJ, PALS1, and PAR3 as well as decreased levels of TJ proteins ZO-1 and ZO-3 in *Crb3^ERΔIEC^* colonoids (Figure 2B). However, the expression level of the AJ protein E-cadherin was unchanged in colonoids in the absence of CRB3 (Figure 2B). Interestingly, discontinuous localization of ZO-1 and mislocalization of E-cadherin were rescued in *Crb3^ERΔIEC^* cells when grown to confluence (Supplemental Figure 2A). Furthermore, to analyze the role of CRB3 in junctional assembly, a calcium switch assay was performed in confluent monolayers of colonoids derived from *Crb3^fl/fl^* and *Crb3^ERΔIEC^* mice. Immunostaining of AJC proteins at 0, 2, and 4 hours following calcium switch revealed reduced and discontinuous junctional localization of ZO-1 in *Crb3^ERΔIEC^* colonoids compared with the control *Crb3^fl/fl^* colonoids (Supplemental Figure 2B). Similar to subconfluent monolayers, E-cadherin staining was diffuse and disrupted along the lateral plasma membrane of *Crb3^ERΔIEC^* colonoids compared with the control *Crb3^fl/fl^* colonoids (Supplemental Figure 2C). However, there was no delay in the targeting of these proteins to the AJC in cells lacking CRB3 (Supplemental Figure 2, B and C). These results suggest that CRB3 regulates the localization and organization of AJC proteins ZO-1 and E-cadherin during junctional assembly in IECs.

In epithelia, organization and contractility of the apical perijunctional actomyosin cytoskeleton are crucial for the formation and maintenance of the AJ (3, 32, 33). Since CRB3 influenced the junctional localization of AJC proteins, we next determined whether CRB3 expression modulates formation of perijunctional actomyosin cytoskeleton. Colabeling of ZO-1 and F-actin in colonoids from *Crb3^fl/fl^* and *Crb3^ERΔIEC^* mice revealed marked changes in the perijunctional F-actin of *Crb3^ERΔIEC^* cells, which displayed a “railroad” appearance along the AJC compared with the control *Crb3^fl/fl^* IECs (Figure 2C, arrows and arrowheads). In *Crb3^ERΔIEC^* cells, this F-actin organization suggested a hypercontractile state (Figure 2C). To minimize interpretation bias, we cocultured colonoids derived from *Crb3^fl/fl^* and *Crb3^ERΔIEC^* mice at a 1:1 ratio and labeled for F-actin, ZO-1, E-cadherin, and PATJ. Based on F-actin morphology, disrupted ZO-1 junctional distribution, and discontinuous junctional localization of PATJ, we delineated the boundary between CRB3-expressing IECs and *Crb3^ERΔIEC^* IECs with a white dotted line in Figure 2D. In *Crb3^ERΔIEC^* cells, ZO-1 and E-cadherin were mislocalized in the AJC, which was associated with disorganization of perijunctional actomyosin belt. Consistent with Figure 2A, the junctional localization of PATJ was substantially reduced in *Crb3^ERΔIEC^* colonoids (Figure 2D). Together, these results support the role of CRB3 in regulating the junctional localization of AJC proteins ZO-1 and E-cadherin and the organization of the perijunctional F-actin cytoskeleton during junctional assembly in IECs.

**Figure 3:**
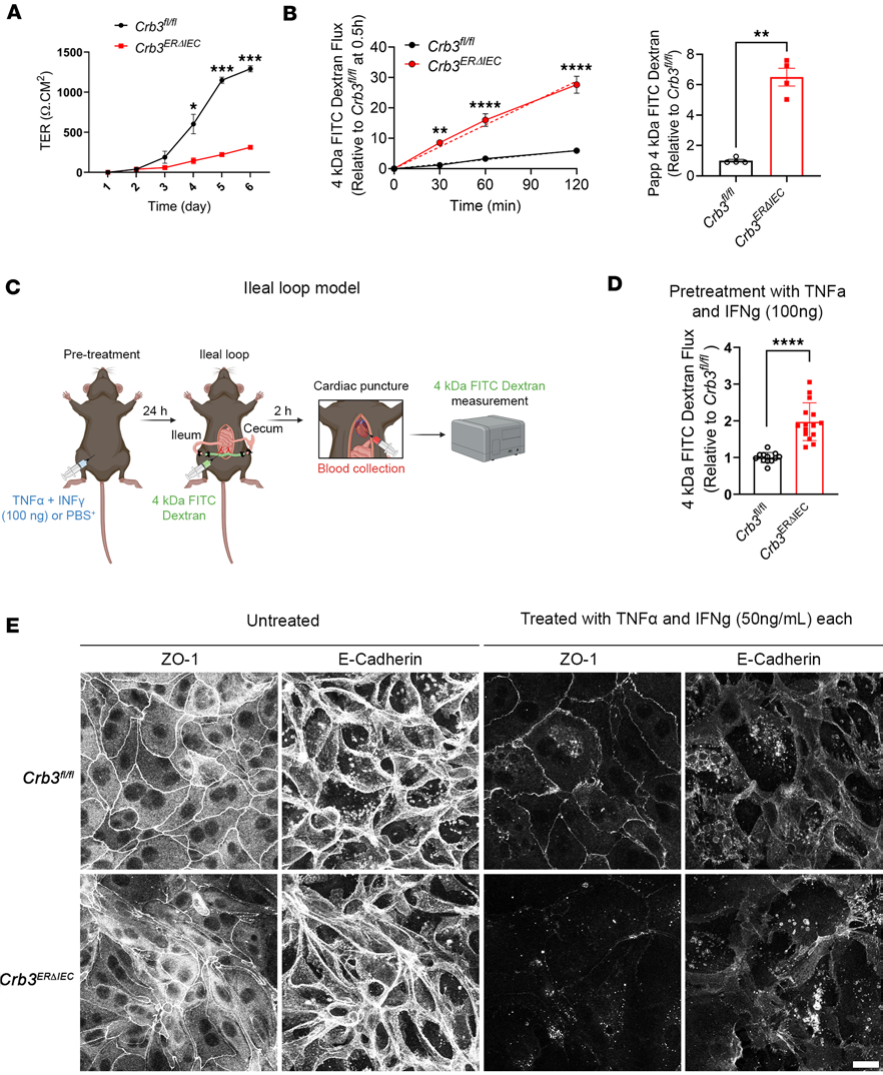
CRB3 regulates the formation of the intestinal epithelial barrier. (**A**) Graph showing establishment of epithelial barrier function that was measured by TER in confluent colonoids from *Crb3^fl/fl^* (shown in black) and *Crb3*^ER*Δ*IEC^ (shown in red) mice grown cultured on Transwells for 6 days (day 1 represents day of isolation). Data are mean ± SD, *n* = 3 experiments each with 6 technical replicates. *P ≤ 0.05, ***P ≤ 0.001 by 2-tailed Student’s *t* test. (**B**) Left: Paracellular flux rate of 4 kDa FITC-dextran across monolayers of primary murine colonoids derived from *Crb3^fl/fl^* and *Crb3*^ER*Δ*IEC^ mice at times indicated on the *x* axis. Data are mean ± SD. *n* = 3 experiments each with 6 technical replicates. **P ≤ 0.01, ****P ≤ 0.0001 by 2-way ANOVA followed by Bonferroni’s post hoc test. Rate of change of TD4 flux was determined by the slope of the linear regression (dotted line: Slope*_Crb3fl/fl_* = 0.05 min^–1^; Slope_Crb3ER*Δ*IEC_ = 0.24 min^–1^). Right: Apparent permeability (*P_app_*) for each individual sample was calculated using the slope values of the linear regression on the left. (**C**) Schematic showing the in vivo ileal loop model in mice to evaluate intestinal epithelial barrier function. (**D**) Intestinal permeability measured by paracellular flux of 4 kDa FITC-dextran in serum of *Crb3^fl/fl^* and *Crb3*^ER*Δ*IEC^ mice 2 hours after surgery in mice pretreated with proinflammatory cytokines (TNF-α and IFN-γ, 100 ng each). Data are mean ± SD. *n* = 3 independent experiments, with at least 3 technical replicates. ****P ≤ 0.0001 by 2-tailed Student’s *t* test. (**E**) Immunofluorescent labeling and confocal images of ZO-1 and E-cadherin in confluent colonoids grown on permeable substrate and treated with proinflammatory cytokines (TNF-α and IFN-γ, 50 ng/mL each) for 24 hours in colonoids derived from *Crb3^fl/fl^* and *Crb3*^ER*Δ*IEC^ mice. Scale bar: 25 μm. *n* = 2 independent experiments.

Defects in formation and maintenance of intestinal epithelial barrier are associated with altered expression and localization of AJC proteins, including ZO-1, E-cadherin, PALS1, and PATJ. Since loss of CRB3 leads to disorganization of the AJC, we evaluated epithelial barrier formation by measuring the transepithelial electrical resistance (TER) and paracellular flux of 4 kDa FITC-dextran (FD4) in colonoid monolayers from *Crb3^fl/fl^* and *Crb3^ERΔIEC^* mice that were cultured on permeable supports. As shown in Figure 3A, loss of CRB3 led to a significant decrease in TER during junction maturation that was evident on day 4, compared with control cells derived from *Crb3^fl/fl^* control colonoids (Figure 3A). Moreover, the FD4 paracellular flux on day 6 of cell culture significantly increased over time in the absence of CRB3 compared with control cells during junctional maturation (Figure 3B). Apparent permeability (*P_app_*) calculated from FD4 paracellular flux was significantly increased (6-fold) in *Crb3^ERΔIEC^* mice (Figure 3B). Altogether, these findings support a key role for CRB3 in promoting epithelial barrier function during junctional maturation in vitro.

Given that CRB3 regulates intestinal epithelial barrier function during junctional assembly in colonoids in vitro, we next assessed its role in junctional remodeling in vivo using an inflammatory model. Proinflammatory cytokines (TNF-α and IFN-γ) modulate intestinal epithelial barrier function by promoting junctional remodeling (34). Thus, to determine the role of CRB3 in junctional remodeling, *Crb3^fl/fl^* and *Crb3^ERΔIEC^* mice were administered proinflammatory cytokines (murine TNF-α and IFN-γ, 100 ng each, intraperitoneally) for 24 hours prior to analysis of intestinal barrier function, which was measured using an intestinal loop model (Figure 3C). Paracellular epithelial permeability was analyzed after administering FD4 into the lumen of a vascularized ileal segment, and its appearance in the serum was measured over a 2-hour period. Serum was collected by cardiac puncture, and FD4 fluorescence intensity was quantified in *Crb3^fl/fl^* and *Crb3^ERΔIEC^* mice and normalized to control *Crb3^fl/fl^* levels (Figure 3C) (35, 36). Following cytokine pretreatment, *Crb3^ERΔIEC^* mice exhibited a significant increase in serum FD4 levels compared with *Crb3^fl/fl^* littermate controls (Figure 3D), indicating enhanced epithelial paracellular flux. However, there was no significant change in the paracellular permeability of *Crb3^fl/fl^* and *Crb3^ERΔIEC^* mice in the absence of proinflammatory cytokines (Supplemental Figure 2D). This is consistent with our finding that the junctional localization of ZO-1 and E-cadherin is comparable between colonoids from *Crb3^fl/fl^* and *Crb3^ERΔIEC^* mice in confluent monolayers that have established junctions (Supplemental Figure 2A). These findings support an important role of CRB3 in junctional assembly and remodeling.

To complement our in vivo analysis of CRB3 in junctional remodeling, colonoids derived from *Crb3^fl/fl^* and *Crb3^ERΔIEC^* mice were cultured as confluent monolayers on permeable supports for 6 days to allow for junction maturation. Monolayers were then treated with 50 ng/mL of murine TNF-α and IFN-γ for 24 hours to induce proinflammatory cytokine–mediated junctional remodeling. Immunofluorescent labeling revealed a marked reduction in ZO-1 intensity and mislocalization of E-cadherin within the AJC of cytokine-treated *Crb3^ERΔIEC^* colonoids compared with *Crb3^fl/fl^* controls (Figure 3E). These results indicate that CRB3 regulates junctional localization of key AJC proteins, ZO-1 and E-cadherin, during epithelial junctional remodeling. Together, these findings demonstrate that CRB3 plays an important role in promoting efficient barrier formation during de novo junctional assembly and cytokine-induced AJC remodeling.

### Loss of CRB3 results in a hypercontractile perijunctional actomyosin ring, driven by enhanced RhoA/ROCK-II signaling.

**Figure 4:**
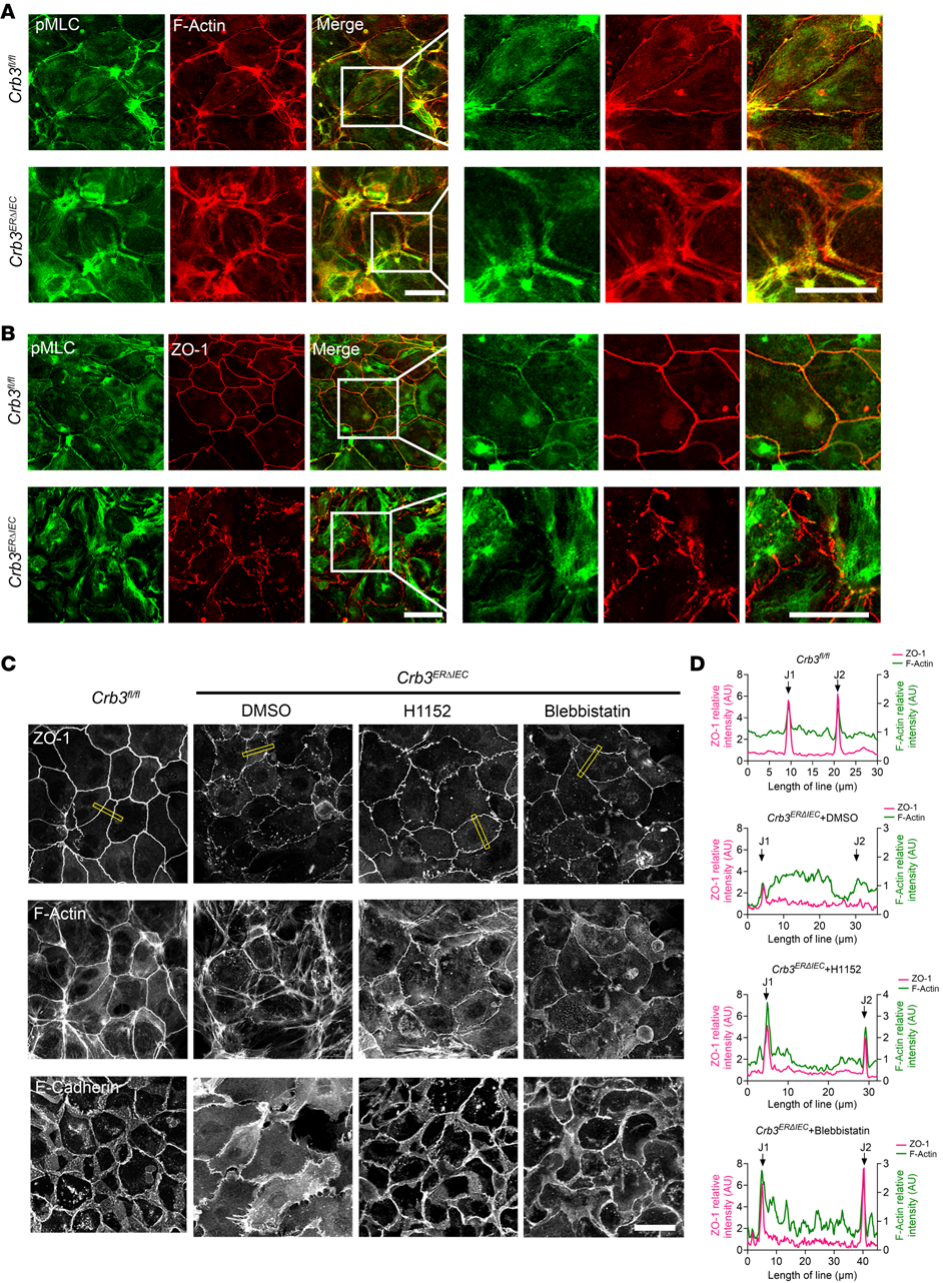
Inhibition of ROCK-II or p-MLC activity largely restores the defects in apical junctional assembly and the organization of the perijunctional actomyosin belt in CRB3-KO IECs. (**A**) Representative confocal images of subconfluent colonoids derived from *Crb3^fl/fl^* and *Crb3*^ER*Δ*IEC^ mice coimmunostained for p-MLC T18/S19 (green) and F-actin (phalloidin, red). Scale bars: 25 μm. *n* = 3 independent experiments, 2 technical replicates each. (**B**) Representative confocal images of subconfluent colonoids derived from *Crb3^fl/fl^* and *Crb3*^ER*Δ*IEC^ mice coimmunostained for p-MLC T18/S19 (green) and ZO-1 (red). Scale bars: 25 μm. *n* = 3 independent experiments, 2 technical replicates each. (**C**) Representative confocal images of perijunctional F-actin (phalloidin) and AJC proteins (ZO-1 and E-cadherin) in *Crb3^fl/fl^* colonoids and *Crb3*^ER*Δ*IEC^ colonoids treated with ROCK II inhibitor (H1152,10 μM), myosin II inhibitor (blebbistatin, 25 μM), or vehicle (DMSO, 0.1%) for 30 minutes. Scale bar: 25 μm. *n* = 3 independent experiments, 2 technical replicates each. (**D**) Graph showing the distribution and relative intensity of ZO-1 (magenta) and F-actin (green) across 20-pixel-thick lines spanning 2 cell-cell junctions. Junctional localizations are denoted by J1 and J2. Yellow box in **C** highlights the cell-cell junctions.

**Figure 5:**
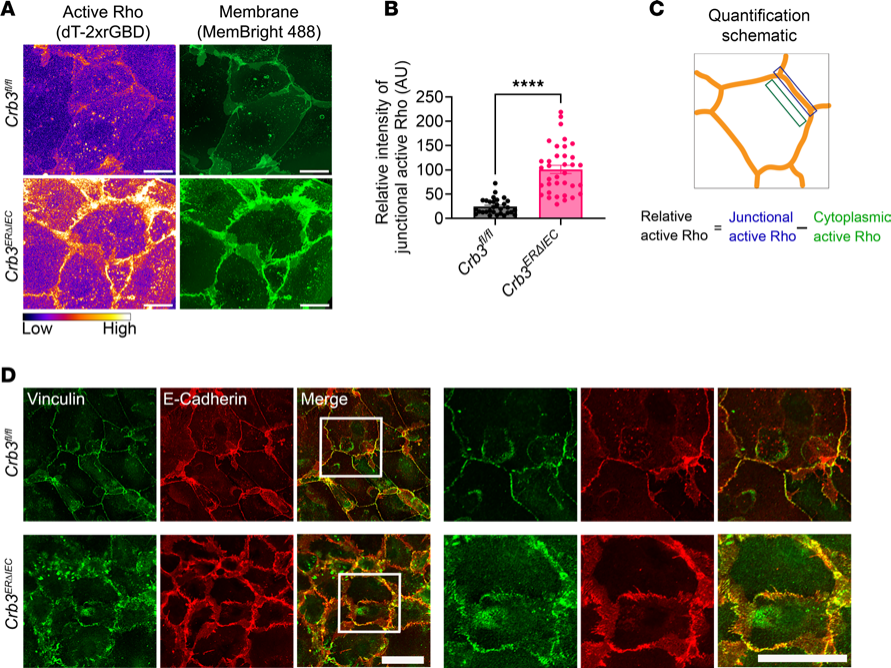
Loss of CRB3 results in increased junctional RhoA activity and increased cell-cell junctional tension during junctional assembly in IECs. (**A**) Representative Airyscan image of subconfluent colonoids derived from *Crb3^fl/fl^* and *Crb3*^ER*Δ*IEC^ mice expressing pLenti-dTomato-2xrGBD (pseudocolored as FIRE LUT) and stained with MemBright 488 plasma membrane dye (green). Images shown are sum of *Z*-projections. Scale bars: 20 μm. (**B**) Graph showing the relative intensity of active Rho at cell-cell junctions normalized to the background signal of every junction. Data are mean ± SEM. Each dot represents an independent junction from 3 independent experiments. ****P < 0.0001 by Mann-Whitney test. *n* = 30 junctions for *Crb3^fl/fl^*; *n* = 36 junctions for *Crb3*^ER*Δ*IEC^. (**C**) Schematic showing the quantification method used to measure the relative intensity of junctional active RhoA. (**D**) Representative confocal images of subconfluent colonoids derived from *Crb3^fl/fl^* and *Crb3*^ER*Δ*IEC^ mice immunostained for Vinculin (green) and E-cadherin (red). White box represents zoomed in region shown on the right. Scale bars: 25 μm. *n* = 3 independent experiments, 2 technical replicates each.

The small GTPase RhoA is a key regulator of the perijunctional actomyosin ring dynamics during epithelial junctional remodeling (37, 38). Upon activation, RhoA (Rho GTP) engages its downstream effector ROCK-II, which promotes actomyosin contractility by phosphorylating myosin light chain II (p-MLCII), thereby generating contractile forces that modulate junctional architecture. Coimmunostaining of F-actin and p-MLCII in subconfluent colonoids from *Crb3^fl/fl^* and *Crb3^ERΔIEC^* mice demonstrated changes in perijunctional actomyosin, which exhibited a “railroad track” appearance, and mislocalization of junctional p-MLCII in *Crb3^ERΔIEC^* colonoids (Figure 4A). Additionally, colocalization of ZO-1 and p-MLCII at TJs was disrupted in *Crb3^ERΔIEC^* cells (Figure 4B). These findings implicate excessive perijunctional actomyosin contractility as a key driver of defective AJC assembly in *Crb3^ERΔIEC^*. To investigate this possibility, subconfluent colonoids derived from *Crb3^ERΔIEC^* mice were treated for 30 minutes with ROCK-II inhibitors (H1152 and Y27632), the myosin II inhibitor blebbistatin, or vehicle control (DMSO). p-MLCII staining confirmed that H1152 and Y-27632 suppressed ROCK-II/myosin light chain phosphatase/p-MLCII signaling, while blebbistatin directly inhibited MLC ATP/ADP turnover rather than MLC phosphorylation (Supplemental Figure 3A). Immunostaining for ZO-1, F-actin, and E-cadherin demonstrated that inhibiting myosin II contractility with these compounds substantially restored general architecture of the perijunctional actomyosin ring (Figure 4C and Supplemental Figure 3B) and the junctional localization of AJC proteins, including TJs (ZO-1) and AJs (E-cadherin) in *Crb3^ERΔIEC^* (Figure 4, C and D, and Supplemental Figure 3B). Line scan across the AJC further demonstrated that mislocalization of ZO-1 and F-actin from the AJC in *Crb3^ERΔIEC^* colonoids was rescued by inhibiting junctional tension using H1152 and blebbistatin (Figure 4D). To examine RhoA activation during junctional assembly, live cell imaging was performed in colonoids derived from *Crb3^fl/fl^* and *Crb3^ERΔIEC^* mice using a localization-dependent biosensor comprised of the rhotekin GTPase-binding domain (rGBD), which detects active RhoA at sites of signaling. As previously described, the fluorescently tagged probe dTomato-2xrGBD specifically associates with the GTP-bound state of RhoA (active RhoA) (39, 40). Live cell imaging of subconfluent colonoids transduced with pLV-dimericTomato-2xrGBD and labeled with the membrane probe MemBright 488 revealed a pronounced increase in junctional accumulation of active RhoA in *Crb3^ERΔIEC^* colonoids compared with controls (*Crb3^fl/fl^* mice) (Figure 5, A–C). Importantly, treatment of *Crb3^ERΔIEC^* colonoids with C3 transferase (C3T), an inhibitor of RhoA activity, significantly reduced junctional dTomato-2xrGBD signal, confirming the probe’s specificity for active RhoA (Supplemental Figure 4A).

To further evaluate the increased mechanical tension at the AJC in cells lacking CRB3, we examined the localization of Vinculin, which is known to be recruited to the AJ by α-catenin under conditions of increased tension (41–43). Coimmunostaining of Vinculin and E-cadherin in subconfluent colonoids derived from *Crb3^fl/fl^* and *Crb3^ERΔIEC^* mice revealed increased Vinculin localization at the AJC in *Crb3^ERΔIEC^* compared with control cells (Figure 5D). To demonstrate antibody specificity, 2 different Vinculin antibodies were used for coimmunostaining with ZO-1 and F-actin (Supplemental Figure 4, B and C). Together, these results demonstrate that CRB3 expression regulates optimal perijunctional actomyosin contractility and maintains appropriate tension at the AJC, a process mediated by RhoA/ROCK-II signaling during IEC junctional assembly.

### PBM and FBD of CRB3A regulate assembly of the AJC and perijunctional F-actin ring in IECs.

The CRB3A C-terminal cytoplasmic tail contains an FBD and PBM. In vertebrates, CRB3 is known to associate with Ezrin, a member of Ezrin-Radixin-Moesin (ERM) protein family, through its FBD (21). ERM proteins have been reported to play a crucial role in organizing the perijunctional actomyosin ring by anchoring it to the cell-cell junctions in epithelial cells (44, 45). To elucidate the mechanism by which CRB3 controls AJC assembly and barrier function, we examined the contribution of the CRB3 FBD and PBM in regulating these processes in primary *Crb3^ERΔIEC^* colonoids.

**Figure 6:**
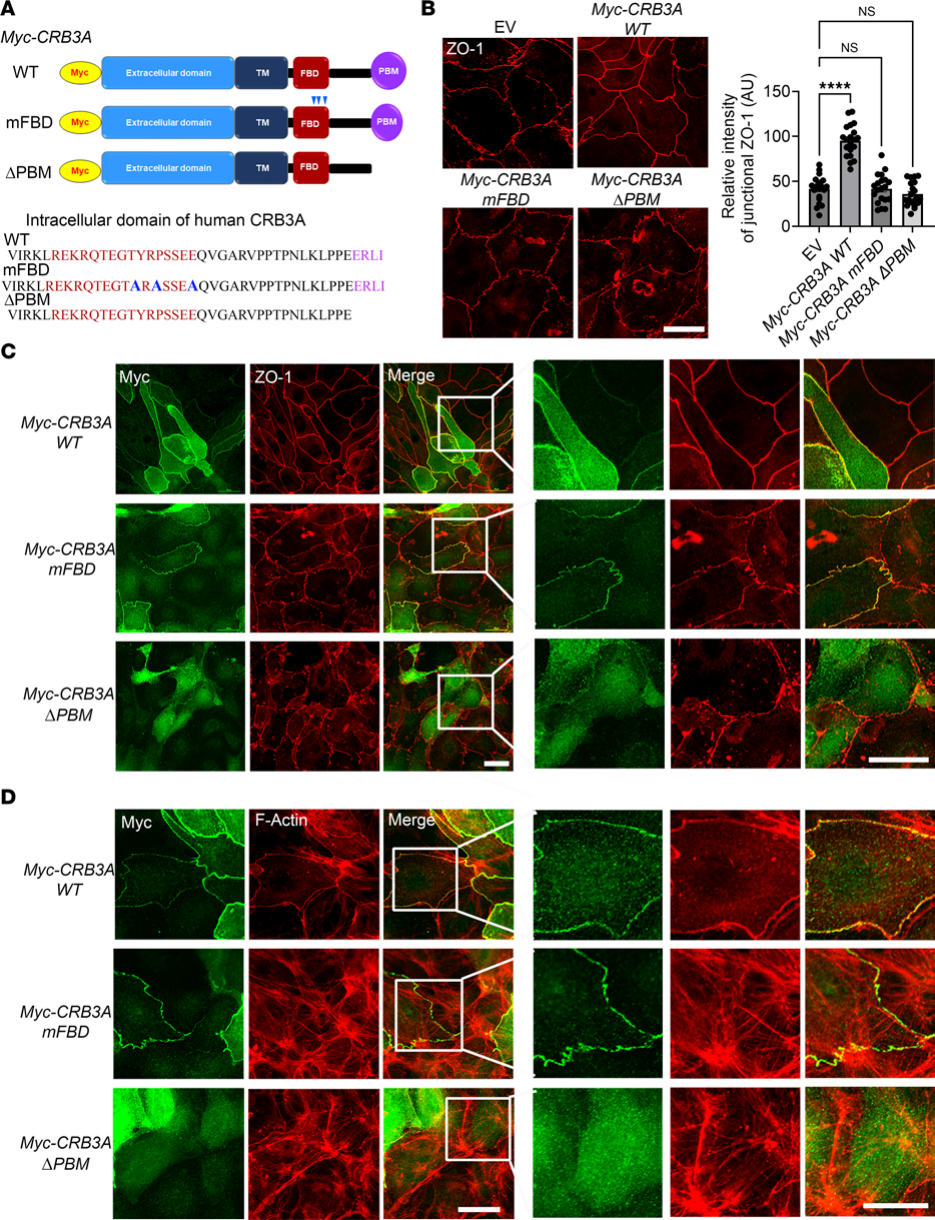
PDZ binding motif (PBM) and FERM binding domain (FBD) of CRB3A control assembly of the AJC and perijunctional F-actin ring in IECs. (**A**) Schematic of WT human CRB3A, CRB3A mutant with 3 amino acid point mutations in FBD (mFBD, blue arrowheads), and CRB3A mutant lacking PBM (ΔPBM). Amino acids in the FBD are shown in brown, mutations shown in blue, and the PBM in purple. (**B**) Left: Immunofluorescent labeling and confocal images of apical junctional protein, ZO-1 (red, during junction assembly in subconfluent colonoids derived from *Crb3*^ER*Δ*IEC^ mice infected with lentiviral CRB3 WT (pLLV Myc-Crb3a WT), ΔPBM motif (pLLV Myc-Crb3a ΔPBM), and mFBD (pLLV Myc-Crb3a FBD mut) constructs. Scale bar: 25 μm. *n* = 3 independent experiments, 3 technical replicates each. Right: Graph showing the relative intensity of ZO-1 at cell-cell junctions normalized to the background signal of every junction. Data are mean ± SEM. Each dot represents an independent junction. ****P < 0.0001 by Brown-Forsythe and Welch’s ANOVA test. (**C**) Costaining of apical junctional protein ZO-1 (red) and Myc-tag (anti-Myc, green) in subconfluent colonoids derived from *Crb3*^ER*Δ*IEC^ mice expressing plasmids described in Figure 4B. Cells stained positive for Myc (green) represent the Crb3-KO murine colonoids expressing the lentiviral Crb3a constructs described in Figure 4A. White box of the zoomed area shown on the right panel. Scale bars: 25 μm. *n* = 3 independent experiments, 2 technical replicates each. (**D**) Costaining of perijunctional F-actin ring (phalloidin, red) and Myc-tag (green) in colonoids derived from *Crb3*^ER*Δ*IEC^ mice described in Figure 4B. White box highlights the zoomed-in area shown in the right panel. Scale bars: 25 μm. *n* = 3 independent experiments, 2 technical replicates each.

WT and mutant human CRB3A tagged with an N-terminal Myc epitope were cloned into a pLentiviral expression vector (pLLV). The constructs illustrated in the schematic in Figure 6A include WT human CRB3A (pLLV Myc-CRB3A WT), a CRB3A mutant with 3 amino acid point mutations in FBD (pLLV Myc-CRB3A mFBD, highlighted in blue), and a CRB3A mutant lacking the ERLI sequence (pLLV Myc-CRB3A ΔPBM). Colonoids harvested from *Crb3^ERΔIEC^* mice were transduced with lentivirus to express CRB3A WT (pLLV Myc-CRB3A WT), CRB3A FBD mutant (pLLV Myc-CRB3A mFBD), or CRB3A ΔPBM mutant (pLLV Myc-CRB3A ΔPBM).

Immunofluorescent labeling for ZO-1 revealed that both *CRB3A* mFBD and *CRB3A* ΔPBM mutants were unable to restore the linear junctional plasma membrane localization of ZO-1, in contrast with the expression of *CRB3A* WT (Figure 6B). Next, we performed coimmunostaining of Myc, ZO-1 (Figure 6C), and F-actin (Figure 6D) in colonoids from *Crb3^ERΔIEC^* mice expressing *CRB3A* WT or *CRB3A* mutants. Myc immunostaining demonstrated junctional localization of CRB3A in *Crb3^ERΔIEC^* colonoids expressing either *CRB3A* WT or *CRB3A* FBD mutant (Figure 6C). However, the CRB3A ΔPBM mutant failed to localize in the junctional plasma membrane in *Crb3^ERΔIEC^* colonoids (Figure 6C), indicating that the CRB3A PBM is required for CRB3 targeting to the epithelial AJC. Furthermore, expression of Myc-*CRB3A* WT in *Crb3^ERΔIEC^* colonoids rescued the junctional localization of ZO-1 (Figure 6C) and perijunctional actomyosin organization (F-actin, Figure 6D), while *CRB3A* mutants (Myc-*CRB3A* mFBD and Myc-*CRB3A* ΔPBM) failed to rescue these parameters (Figure 6, C and D). Collectively, our results demonstrate that CRB3A is sufficient to rescue AJC protein and perijunctional actomyosin in *Crb3^ERΔIEC^* colonoids, and the CRB3A PBM is essential for its proper junctional localization. Moreover, both the CRB3A FBD and PBM control apical junctional assembly and the organization of the perijunctional actomyosin ring in IECs.

### NF2 interacts with the CRB3 FBD during AJC assembly in IECs.

**Figure 7:**
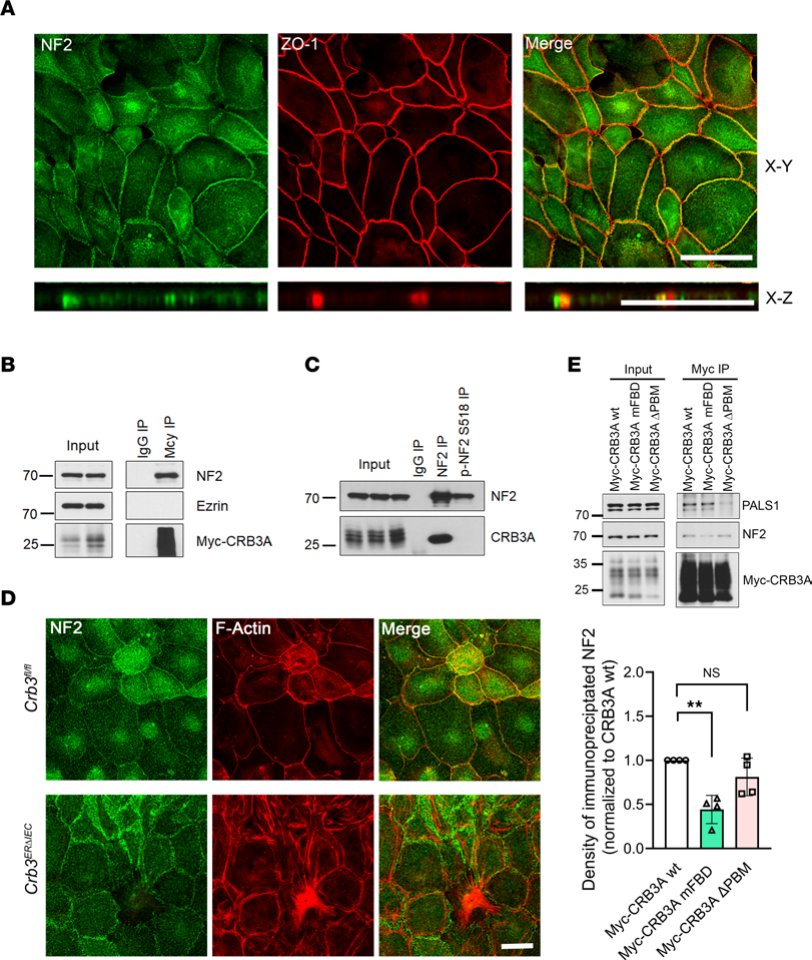
NF2 interacts with CRB3A in IECs and regulates epithelial junctional assembly. (**A**) Top: Representative confocal image of primary mouse colonoids stained for NF2 and TJ protein ZO-1. Images shown are sum of *Z*-projections. Bottom: *X*-*Z* projections showing the localization along the apical-basal polarity. Scale bars: 25 μm. *n* = 3 independent experiments, 2 technical replicates each. (**B**) Immunoprecipitation of CRB3A in SKCO-15 IECs expressing Myc-Crb3a WT followed by Western blotting for NF2, Ezrin, and Myc-tag. Myc-Crb3a immunoprecipitates NF2, but not Ezrin. Input shows equal loading between samples and IgG immunoprecipitation serves as a negative control; *n* = 3 independent experiments. (**C**) Immunoprecipitation of endogenous NF2 and p-NF2 (S518) in SKCO-15 IECs followed by Western blotting for CRB3A; *n* = 3 independent experiments. (**D**) Representative confocal images of immunostained proteins showing the junctional localization of NF2 (green), F-actin (red), and merge in subconfluent murine colonoids derived from *Crb3^fl/fl^* and *Crb3*^ER*Δ*IEC^ mice. Scale bar: 25 μm. *n* = 3 independent experiments, 2 technical replicates each. (**E**) Top: Immunoprecipitation of anti-Myc antibody in SKCO-15 IECs expressing Myc-Crb3a WT, Crb3a FBD mutant, or Crb3a PBM followed by Western blotting for NF2, PALS1, and Myc-tag. Decreased binding of NF2 in Crb3a FBD mutant, while Crb3a PBM did not associate with PALS1. Bottom: Graph showing densitometry of NF2 immunoblot relative to loading control and normalized to SKCO-15 IECs expressing Myc-Crb3a WT. Data are mean ± SD. *n* = 4. Each dot represents an independent experiment. **P ≤ 0.01 by ordinary 1-way ANOVA with Dunnett’s multiple-comparison test.

Our results demonstrate that the cytoplasmic CRB3 FBD regulates AJC assembly and organization of the perijunctional F-actin ring. In *Drosophila*, CRB interacts with Expanded (Ex) and 2 other FERM-containing proteins, Yurt and Moesin, via the CRB cytoplasmic FBD to regulate the Hippo signaling pathway (14, 18, 46). In humans, CRB3 binds to Ezrin and regulates the morphogenesis of intestinal villi and microvilli (25, 47). While Ezrin is localized in the apical domain of IECs, we did not detect its presence at the TJ (Supplemental Figure 5). Given the exclusive localization of Ezrin in the apical domain, it is likely that other ERM proteins interact with colonic epithelial CRB3 to regulate AJC assembly and perijunctional actomyosin organization. NF2, an ERM family member, is known to localize at the AJC and interact with TJ protein Angiomotin (48), as well as AJ protein α-catenin in epithelial cells (45). Indeed, we observed NF2 colocalization with ZO-1 in primary mouse colonoids (Figure 7A).

To investigate whether CRB3 is part of a protein complex with NF2, we evaluated the potential association between CRB3 and NF2. Coimmunoprecipitation of CRB3A with NF2 was performed using the model IEC line SKCO-15 expressing Myc-tagged full-length *CRB3A* (Myc-*CRB3A* WT). Anti-Myc antibodies coimmunoprecipitated with endogenous NF2, but not Ezrin (Figure 7B). Additionally, endogenous NF2 coimmunoprecipitated with endogenous CRB3 in SKCO-15 cells (Figure 7C). But the inactive form of NF2 (49), p-NF2S518, which is shown to be in the cytoplasm, failed to pull down CRB3 in SKCO-15 cells (Figure 7C). This suggests that the CRB3-NF2 interaction is restricted to the junctions. Furthermore, immunostaining of subconfluent colonoids from *Crb3^fl/fl^* and *Crb3^ERΔIEC^* mice revealed that NF2 localization at the AJC was reduced in the absence of CRB3 (Figure 7D). However, expression levels of total cellular NF2 protein were comparable between colonoids from *Crb3^fl/fl^* and *Crb3^ERΔIEC^* mice (Supplemental Figure 6A). Together, our results demonstrate that CRB3 interacts with active NF2, and this interaction is critical for the proper localization of NF2 at AJCs.

Next, we assessed the interaction of NF2 with the FBM and PBM of CRB3 by performing coimmunoprecipitation experiments in SKCO-15 cells expressing Myc-tagged *CRB3A* WT and Myc-tagged *CRB3A* mutants. Our results indicate that while anti-Myc antibodies coimmunoprecipitated endogenous NF2 with Myc-CRB3A WT and Myc-CRB3A ΔPBM, the interaction was significantly reduced with Myc-CRB3A mFBD (Figure 7E). Additionally, the interaction between CRB3 and PALS1 was reduced only in cells expressing the Myc-CRB3A ΔPBM mutant, consistent with previous studies that showed PALS1 interacts with the PBM of CRB3 (7) (Figure 7E). These results collectively demonstrate that NF2 interacts with the FBD of CRB3, and that the junctional localization of NF2 is modulated by CRB3.

### NF2 KD phenocopies the effects of CRB3 deletion, disrupting AJC assembly and compromising IEC barrier function.

**Figure 8:**
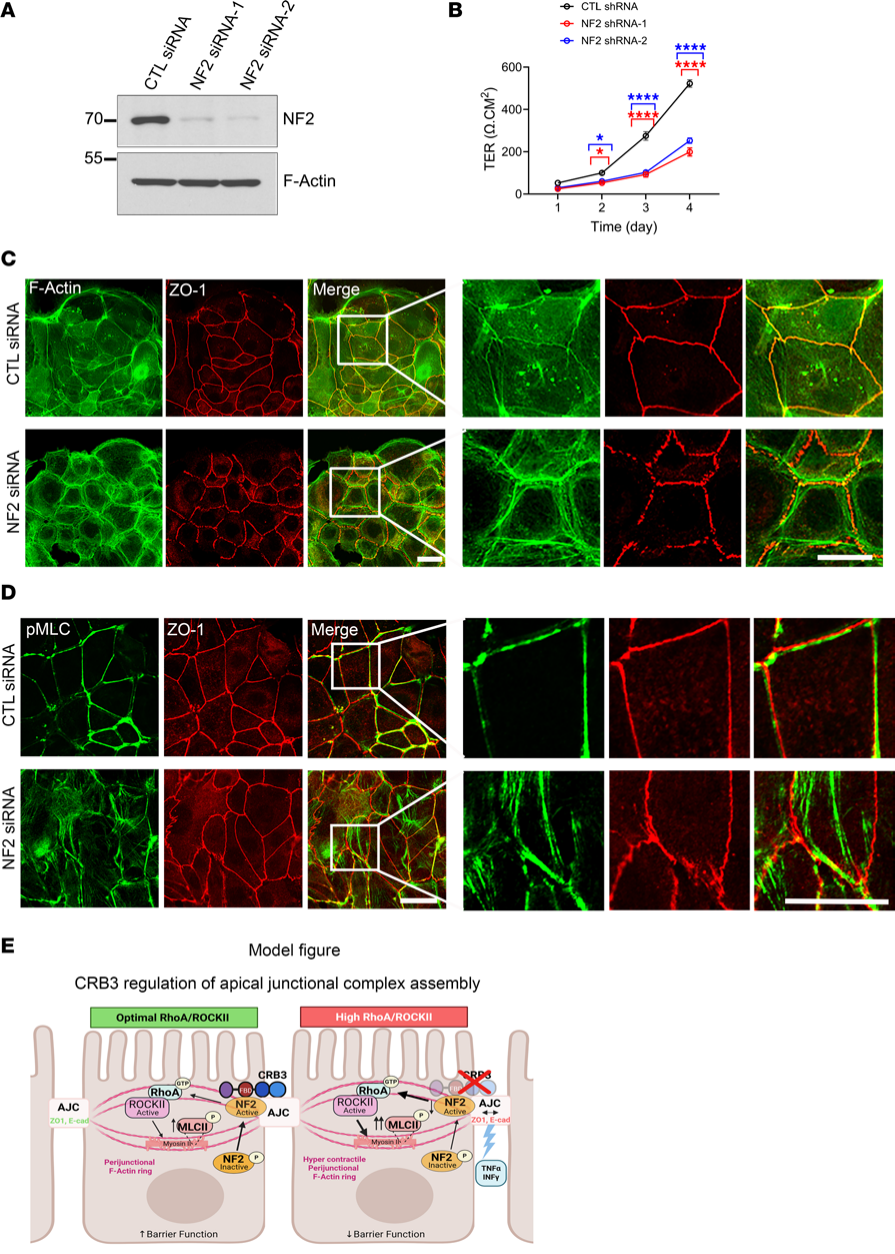
Loss of NF2 mimics the defects seen in CRB3-deficient IECs, including impaired apical junctional assembly, disorganization of the perijunctional actomyosin belt, and compromised epithelial barrier function. (**A**) Representative immunoblot showing the efficiency of NF2 knockdown in SKCO-15 IECs transfected with control scrambled or NF2 siRNA, with F-actin as a loading control. *n* = 3 independent experiments. (**B**) Graph showing TER of SKCO-15 cells with siRNA-mediated knockdown of NF2 or scramble control in cells cultured as monolayers for 4 days. Data are mean ± SD, *n* = 3 experiments each with 6 technical replicates. *P ≤ 0.05; ****P ≤ 0.0001 by 2-way ANOVA. (**C**) Representative perijunctional F-actin organization (phalloidin, green) and tight junction protein ZO-1 (red) localization in subconfluent SKCO-15 cells with siRNA knockdown of NF2 or control scramble siRNA for 48 hours. White box highlights an area that is enlarged in the right panel. Scale bars: 25 μm. *n* = 3 independent experiments, 3 technical replicates each. (**D**) Immunostaining of junctional myosin II (p-MLC [T18/S19], green) and the tight junction protein ZO-1 (red) in subconfluent SKCO-15 with NF2 siRNA–mediated knockdown or control scramble siRNA for 48 hours. White box highlights the zoomed-in area shown in the right panel. Scale bars: 25 μm. *n* = 3 independent experiments, 3 technical replicates each. (**E**) Model figure illustrating the role of CRB3 and its binding partner NF2 in regulating perijunctional actomyosin belt and barrier function via RhoA/ROCK-II signaling during apical junctional complex assembly/remodeling.

NF2 plays an important role in anchoring the perijunctional actomyosin ring to the plasma membrane (45). Given our findings that CRB3 interacts with active NF2 in IECs, we investigated whether NF2, like CRB3, contributes to AJC assembly, organization of the perijunctional actomyosin ring, and epithelial barrier function. NF2 was knocked down in model IECs, SKCO-15, using 2 independent siRNAs targeting NF2. As shown in Figure 8A and Supplemental Figure 6B, both NF2 siRNAs downregulated NF2 expression compared with the scrambled control siRNA. Barrier function was analyzed by measuring the TER of epithelial cells cultured on permeable supports for 4 days. NF2 KD resulted in a significant reduction in TER development relative to control cells, indicating impaired barrier function (Figure 8B).

Coimmunostaining of F-actin and ZO-1 in control and NF2-KD cells revealed that NF2 depletion disrupted the organization of perijunctional actomyosin ring and resulted in discontinuous ZO-1 staining at TJs, which is a phenotype that closely resembles those observed in *Crb3^ERΔIEC^* (Figure 8C). In addition to the organization of the perijunctional actomyosin ring, contractility of actomyosin regulates AJC assembly and epithelial barrier function (1, 3). Cytoskeletal contractility is regulated by phosphorylation of MLCII at threonine 18 and serine 19 (p-MLCII^T18/S19^). Coimmunostaining for p-MLCII^T18/S19^ and ZO-1 in NF2-KD cells revealed disorganization and displacement of p-MLCII from the AJC compared with control cells, which is consistent with a hypercontractile actomyosin ring (Figure 8D). Similar to *Crb3^ERΔIEC^* cells, treatment with the ROCK-II inhibitor (H1152) and a myosin II inhibitor (blebbistatin) largely restored both ZO-1 distribution and F-actin ring organization in NF2-KD IECs (Supplemental Figure 7, A and B). Overall, these findings demonstrate that NF2 regulates perijunctional actomyosin organization, AJC assembly, and the establishment of epithelial barrier function, analogous to IEC CRB3. Given the interaction of CRB3 with active NF2, these data suggest that CRB3A and NF2 function cooperatively to regulate actomyosin organization and AJC assembly in IECs (Figure 8E).

## Discussion

Epithelial morphogenesis is a fundamental process that shapes the structure and function of epithelial tissue during development and tissue homeostasis. Apical cell-cell junctions help establish cell polarity and maintain a functional epithelial barrier, both of which are critical for tissue organization and function.

Previous studies have established a role for CRB3 in regulating intestinal epithelial morphogenesis. However, the specific mechanisms by which CRB3 regulates intestinal epithelial barrier function in vivo or in primary colonic epithelial cells (colonoids) is unclear, largely due to premature lethality associated with germline deletion of *Crb3* in mice (21). In this study, we generated a tamoxifen-inducible IEC-specific *Crb3*-KO mouse model (*Crb3^ERΔIEC^*). The small intestine and colonic mucosa of *Crb3^ERΔIEC^* mice displayed normal architecture indistinguishable from that of *Crb3^fl/fl^* littermates. This finding suggests that villus fusion and disrupted apical membranes seen in mice with germline mutations of *Crb3* likely result from dysregulated embryonic development rather than postnatal maintenance. To investigate the role of CRB3 in junctional assembly, we generated CRB3-null colonoids from *Crb3^ERΔIEC^* mice. These colonoids were monitored during barrier maturation and compared to WT *Crb3^fl/fl^*, offering insights beyond those obtained from immortalized or cancer cell lines (7, 50). Primary colonoids provide a physiologically relevant model system that better recapitulates in vivo epithelial architecture, polarity, and junctional dynamics, allowing for more accurate analysis of CRB3’s role in AJC assembly and barrier function. Using this inducible mouse model and primary colonic epithelial cells, our findings demonstrate that CRB3 is a key regulator of apical intercellular junctional assembly and junctional remodeling in the intestine (Figure 8E).

We found that CRB3 localizes in the apical membrane and AJCs of both mouse and human colonic epithelia, in agreement with previous observations in MDCK cells (51). Establishment of epithelial barrier function requires tightly controlled spatiotemporal assembly of intercellular junctions that include the TJ, AJ, and associations with the apical perijunctional actomyosin cytoskeleton. Defects in formation and remodeling of the AJC during mucosal inflammation result in a compromised intestinal epithelial barrier, a hallmark of chronic mucosal inflammatory diseases, including ulcerative colitis and Crohn disease (52). We identified epithelial barrier compromise that was associated with changes in AJC protein localization both in vivo and in vitro in *Crb3^ERΔIEC^* mice during junctional assembly and remodeling. These changes in AJC localization were rescued in *Crb3^ERΔIEC^* colonoids upon reaching confluence, and increased intestinal permeability was not evident in *Crb3^ERΔIEC^* mice in the absence of proinflammatory cytokines. Moreover, the colonic mucosa of *Crb3^ERΔIEC^* and *Crb3^fl/fl^* mice displayed intact colonic mucosal architecture under steady-state conditions. Together, these findings indicate that CRB3 is dispensable for maintenance of mature junctions but is required for efficient barrier establishment during de novo junctional assembly and cytokine-induced remodeling.

Changes in barrier function were also associated with disorganization of the apical perijunctional actomyosin ring that exhibited a “railroad” pattern suggestive of increased actomyosin tension at the AJC, as evidenced by increased expression of both p-MLCII and Vinculin at the AJC. These changes imply that CRB3 regulates localization of AJC proteins while also playing an important role in organizing the actin cytoskeleton, an essential element in the control of epithelial integrity (21, 33, 50, 53).

Spatiotemporally controlled regulation of F-actin polymerization and myosin II–mediated contraction of the apical actomyosin ring is critical for AJC assembly and establishment of epithelial barrier function. The small GTPase RhoA, in its active form, functions as a master regulator of organization and contractility of the apical actomyosin ring. Several studies have demonstrated that a balance of RhoA signaling is essential for the establishment and remodeling of the AJC and epithelial barrier function (1, 38, 54, 55). Our study revealed that the loss of CRB3 or NF2 in IECs increases F-actin and phosphorylated myosin II density in the perijunctional actomyosin ring. Inhibiting myosin II hypercontractility with ROCK-II inhibitors (Y27632, H1152) or myosin II ATPase inhibitor (blebbistatin) in *Crb3^ERΔIEC^* colonoids restored the perijunctional actomyosin ring organization and AJC protein localization. Live cell imaging revealed higher RhoA activity at apical junctions in *Crb3^ERΔIEC^* colonoids compared with controls. Additionally, *Crb3^ERΔIEC^* cells exhibited higher junctional tension, indicated by Vinculin localization at the AJC (42, 43). Our findings suggest that CRB3 loss enhances RhoA activity at apical junctions, leading to increased actomyosin contractility via ROCK-II–mediated MLCII phosphorylation and increased junctional tension. Changes in F-actin polymerization may also be driven by actin-binding proteins such as formins, which act downstream of RhoA signaling (3). The mechanism linking CRB3 loss to increased RhoA activation at apical junctions is unclear but may involve dysregulation of RhoA modulators such as RhoGEF (p114RhoGEF) or RhoGAP (ARHGAP29), both of which are associated with TJs and are known to be regulated by CRB3 interacting partners, PALS1 and PATJ (51, 56). Further investigation is required to delineate the signaling pathways involved and to determine the functional relevance of these candidate regulators in the context of CRB3. Interestingly, CRB3 promotes activation of RhoA by interacting with RhoGEF proteins (such as Cysts in *Drosophila*) to drive apical constriction (57); conversely, loss of CRB3 reduces active Rho, resulting in decreased cell migration and contractility in squamous cell carcinomas (58). Together, such observations highlight the complex regulatory role of CRB3 in balancing RhoA activity and actomyosin contractility in a context-dependent manner.

The transmembrane CRB3 protein has been reported to interact with PALS1-PATJ and PAR3-PAR6-aPKC protein complexes, contributing to establishment and maintenance of apical-basal polarity in epithelial cells. We observed that the absence of CRB3 in colonoids led to a notable reduction in PALS1, PATJ, and PAR3 during junctional assembly. These findings suggest that in addition to its established role in controlling epithelial polarity, CRB3 also contributes to junctional assembly (12, 50, 59). Previous studies identified that CRB3 associates with PALS1 and PAR6 through the PBM, to establish apical-basal polarity and mature TJs in model epithelial cells (12, 26). Specifically, KD of PALS1 or PATJ results in a leaky epithelial barrier associated with decreased junctional F-actin and discontinuous ZO-1 localization at TJs in epithelial cell lines (8, 60). We observed discontinuous ZO-1 localization that was associated with disorganization of the perijunctional actomyosin ring in primary murine IECs expressing the *CRB3* ΔPBM. Analogous to a *CRB3* ΔPBM mutant, putative mutations in the *CRB3* mFBD mutant failed to rescue uniform junctional localization of a TJ protein (ZO-1) and organization of perijunctional actomyosin rings. These findings suggest that both the FBD and PBM of CRB3 are required for the appropriate assembly and organization of the AJC and perijunctional actomyosin ring.

CRB3 associates with several proteins containing FERM domains, such as members of the ERM family through the FBD. In *Drosophila*, Crumbs interacts with the FERM domain protein Expanded to regulate Hippo signaling and can also bind Moesin and Yurt to influence junctional stability (18, 46, 61–64). However, the specific binding partners for the mammalian CRB3 FBD remain to be fully elucidated. CRB3B has been shown to interact with FERM domain proteins like Ezrin to promote TJ formation (25). Studies in mice have demonstrated that loss of Ezrin phenocopies the abnormal morphogenesis of intestinal villi and microvilli seen in CRB3 mutants, suggesting that CRB3 may regulate intestinal morphogenesis during embryonic development through an Ezrin-dependent pathway (44, 47). However, our data indicate that in IECs from adult mice, CRB3A does not interact with Ezrin during junction formation, implying that another ERM family member may mediate this function in AJCs.

NF2, a member of the ERM protein family, has been shown to localize to both TJs and AJs in MDCK cells (48). It forms a complex with PALS1-PATJ at TJs (48) and directly interacts with α-catenin at AJs, thereby linking AJs to the underlying perijunctional actomyosin cytoskeleton in epithelial cells (45). Consistent with this, our data show that while Ezrin is restricted to the apical membrane, NF2 localizes to the AJC in a pattern similar to CRB3. Biochemical pull-down assays revealed that CRB3 specifically interacts with the active (nonphosphorylated) NF2 via its FBD, whereas the phosphorylated, inactive NF2 (p-NF2, serine 518) does not associate with CRB3 (65–67). These findings suggest that CRB3’s interaction with NF2 contributes to apical junctional assembly, potentially through activation of Hippo signaling pathways in IECs (16, 59, 68). Consistent with this, NF2 KD in IECs impaired barrier formation, as indicated by a significant reduction in TER, disrupted organization of the perijunctional actomyosin ring, and discontinuous ZO-1 localization at TJs. These defects closely phenocopy those observed in *Crb3^ERΔIEC^* colonoids and in colonoids expressing a mutant *CRB3* FBD, highlighting the role of the CRB3-NF2 interaction in apical junctional assembly and the establishment of epithelial barrier function.

Interestingly, our study found that while a CRB3A FBD mutant significantly decreased the CRB3-NF2 interaction, the association with PALS1 was not altered. This observation aligns with previous findings that CRB3 associates with the PALS1-PATJ complex via the PBM domain. Despite the ability of the CRB3 FBD to interact with the PALS1-PATJ complex, expression of this mutant did not rescue organization of the perijunctional actomyosin ring and ZO-1 localization within TJs. Notably, CRB3 can associate with NF2 indirectly via PAR3 (45, 69) or PATJ (48, 70, 71) through the CRB3 PBM. Thus, CRB3A appears to engage NF2 through 2 mechanisms: directly via the FBD and indirectly via the PBM. This dual mode of interaction likely explains why mutants lacking either the FBD or PBM domains are unable to rescue the junctional localization of ZO-1 and the perijunctional actomyosin structure. Collectively, these findings underscore the intricate network of CRB3 interactions mediated by its FBD and PBM, which are essential for organizing the perijunctional actomyosin ring and facilitating assembly of the AJC in IECs.

In summary, our findings highlight the critical role of CRB3 in orchestrating the spatiotemporal organization of apical junctions and the actomyosin cytoskeleton during junctional assembly in IECs (Figure 8E). Through its interaction with NF2, CRB3 modulates RhoA signaling and myosin II–dependent contractility to fine-tune perijunctional tension, which are key processes required for coordinated AJC assembly and epithelial barrier establishment. Importantly, the role of CRB3 is context dependent, being dispensable once junctions are established but essential during dynamic phases of junctional assembly/remodeling. This function is particularly relevant in pathological states such as mucosal inflammation, including IBD, where cytokine-induced junction remodeling contributes to epithelial barrier dysfunction. Our results suggest that CRB3, through interactions with NF2 and regulation of actomyosin dynamics, may act as a checkpoint in epithelial barrier restoration. Although the roles of CRB3 and NF2 in IBD are not well defined, our study reveals a previously unrecognized mechanism by which polarity proteins regulate junctional remodeling and barrier formation. These findings provide a conceptual framework for targeting epithelial repair pathways in mucosal inflammatory diseases.

## Methods

### Sex as a biological variable.

Our study examined male and female animals, and similar findings are reported for both sexes.

### Antibodies and reagents.

All antibodies and reagents used in this study are summarized in Supplemental Table 1.

### Mice.

*Crb^ERΔIEC^* and littermate control *Crb3^fl/fl^* mice were generated by breeding *Crb3^fl/fl^* (21) and Villin^ERT2-Cre^ in-house at the University of Michigan in Ann Arbor. Eight-week-old *Crb3^ERΔIEC^* and control *Crb3^fl/fl^* mice were injected intraperitoneally with 1 mg/100 μL of tamoxifen (Sigma-Aldrich, T5648) for 5 consecutive days followed by a period of 30 days to rest. Mice were kept under specific pathogen–free (SPF) conditions with ad libitum access to normal chow and water.

### IECs and murine colonoid monolayer culture.

SKCO-15 and primary murine colonoids were cultured as previous described (72). Briefly, colons were harvested from *Crb3^ERΔIEC^* and *Crb3^fl/fl^* mice and treated with tamoxifen and rested for 30 days. Harvested colons were subjected to chemical disruption using 50 mM EDTA in 1× calcium- and magnesium-free PBS for 40 minutes followed by mechanical shaking in shaking buffer (43.3 mM sucrose and 54.9 mM sorbitol in 1× calcium- and magnesium-free PBS) to isolate colonic crypts. Colonic crypts were plated directly on culture plates coated with human collagen type IV (33 μg/mL; Sigma-Aldrich, 5533) and rh-Laminin (Thermo Fisher Scientific, A29248). Murine colonoids were maintained until the desired confluence in LWRN conditioned media supplemented with 50 ng/mL human EGF (R&D Systems, 236-EG) and antibiotics-antimycotic (Corning, 30-003-Cl).

### Plasmids.

siRNA targeting human NF2 and control (scrambled) siRNA were purchased from Thermo Fisher Scientific (Silencer Select siRNA, 4392421; Silencer Select negative control siRNA, 4390844). Lentiviral plasmids Lentilox-RSV-Myc-Crb3a-CMV-GFP-puro-VSVG-WT and mutants were made at the Vector Core of the University of Michigan. Lentiviral plasmid expressing active RhoA probe, pLV-dimericTomato-2xrGBD was a gift from Dorus Gadella (Addgene plasmid 1760980).

### siRNA transfection, exogenous Crb3 transfection, and lentiviral infections.

Human model IECs (SKCO-15) and murine primary colonic epithelial cell monolayers were cultured either on Transwell permeable supports (Corning) or on tissue culture plastic chamber slides as described above. KD of Crb3 or NF2 in SKCO-15 cells was established by transfecting siRNA specifically targeting Crb3 or NF2 using Lipofectamine RNAiMAX (Thermo Fisher Scientific, 13778150). A scrambled and non-silencing siRNA was used to generate control cells. For ectopic expression of Myc-tagged Crb3, SKCO-15 cells were transfected with human pSEC Myc-*Crb3* WT and mutant constructs using Lipofectamine 3000 (Thermo Fisher Scientific, 3000001); colonoids derived from *Crb3^ERΔIEC^* and *Crb3^fl/fl^* mice were infected with lentivirus expressing vector pLLV human Crb3a WT and mutants using 8 μg/mL of polybrene (MilliporeSigma, TR-1003).

### Immunofluorescence.

SKCO-15 cells and murine colonoids were cultured on plastic chamber slides were fixed with 4% paraformaldehyde (PFA) and permeabilized with 0.2%–0.5% Triton X-100 for 10 minutes. Monolayers were blocked with 3% goat or donkey serum in PBS (with calcium and magnesium) with 0.05% Tween 20 (blocking buffer) for 30 minutes. Primary antibodies were diluted in blocking buffer and cells were incubated overnight at 4°C. Cells were washed with PBS with 0.05% Tween 20 and fluorescently labeled secondary antibodies were diluted in blocking buffer followed by incubation for 1 hour at room temperature. Cells were washed and mounted in Prolong Gold antifade agent (Thermo Fisher Scientific, P36930). Frozen colonic tissue sections (6–8 μm) from *Crb3^ERΔIEC^* and *Crb3^fl/fl^* mice were fixed with 4% PFA, permeabilized with 0.5% Triton X-100, and immunolabeling performed as described above. High-resolution confocal fluorescence images were captured using a Nikon A1 confocal microscope in the Microscopy and Image Analysis Laboratory Core at the University of Michigan.

### Immunoprecipitation and Western blotting.

For immunoprecipitation studies, SKCO-15 naive or SKCO-15 transfected with exogenous Myc-Crb3 WT and mutants were harvested in relaxation lysis buffer (10 mM HEPES, 10 mM NaCl, 3.5 mM MgCl_2_, 100 mM KCl, 1% octyl glucoside) supplemented with a cocktail of protease and phosphatase inhibitors. Epithelial lysates were centrifuged, and supernatants collected. Supernatants were precleared with 50 μL of 50% protein A/G agarose beads (Thermo Fisher Scientific, 20421) for 1 hour followed by incubating with 5 μg/mL antibodies or control IgG rotated overnight at 4°C. Immune complexes were precipitated with 25 μL of protein G Dynabeads (Thermo Fisher Scientific, 1003D) for 4 hours. Immunoprecipitated complexes were washed before boiling in 2× NuPAGE LDS Sample Buffer (Thermo Fisher Scientific, NP0007). Immunoprecipitates and input were then subjected to SDS-PAGE followed by immunoblotting.

### Permeability assay.

Colonoids derived from *Crb3^ERΔIEC^* and *Crb3^fl/fl^* mice were grown on Transwell permeable supports (0.4 μm pore size, Corning, 3460) to confluence. TER to passive ion flow was measured continuously daily using an EVOMX voltmeter (World Precision Instruments) for 6 days. For FITC-dextran flux experiments, colonoids from *Crb3^ERΔIEC^* and *Crb3^fl/fl^* mice were grown on Transwell permeable supports until TER of *Crb3^fl/fl^* colonoids reached 600 Ω•cm^2^. After upper and lower Transwell compartments were washed twice with HBSS and pyruvate buffer (10 mM HEPES, pH 7.4, 1 mM sodium pyruvate, 10 mM glucose, 3 mM CaCl_2_, 145 mM NaCl_2_) was placed in both of upper and lower compartments for 1 hour at 37°C. A freshly prepared solution of 4 kDa FITC-dextran dissolved in pyruvate buffer was added to the top chamber of the Transwells and incubated with gentle shaking for 2 hour at 37°C. Samples from the bottom chamber of the Transwells were collected at the indicated time points, and the fluorescence intensity was measured with a fluorescence plate reader (BioTek). The fluorescence detected from the bottom chamber was expressed as the flux of FITC-dextran over time. This was used to calculate the change in fluorescence per minute (slope value) through linear regression. The slope values were then applied to determine the apparent permeability using the following formula: *P_app_* = (*dQ*/*dt*)/(*A* × *C*_0_), where *dQ/dt* is the rate of permeation (the amount of substance/fluorescence crossing per unit time, represented by the slope value), *A* is the surface area of the membrane, and *C*_0_ is the initial concentration of the substance on the donor side. Data were normalized to control values.

### Ileal loop model for in vivo permeability measurement.

Intestinal permeability was assessed in *Crb3^ERΔIEC^* and *Crb3^fl/fl^* mice as previously described (35, 36). In brief, a 2 cm segment of the ileum in surgically exteriorized and isolated while maintaining vascular supply. The ileal loop was gently flushed with warm 1× HBSS to remove contents. Using a 1 mL syringe, 200 μL of 4 kDa FITC-dextran was injected into the lumen of the exteriorized ileum. After 2 hours of incubation of the mice in a temperature-controlled anesthesia chamber, blood was collected by cardiac puncture. Equal volumes of samples were transferred to black 96-well plate and FITC was measured using a fluorescence plate reader (excitation 490 nm, emission 520 nm).

### Calcium switch.

Monolayers of murine colonoids derived from *Crb3^ERΔIEC^* and *Crb3^fl/fl^* mice were cultured on Transwell permeable supports to confluence in LWRN media. For calcium switch assay, LWRN medium was removed, and cells were washed twice with 1× PBS without calcium and magnesium. Colonoids were incubated overnight in DMEM without calcium (Gibco, 21068-028) supplemented with 10% dialyzed FBS (Gibco, A33820-01), 15 mM HEPES, 10 mM glutamine, and penicillin/streptomycin. On the following day, colonoids were switched to complete LWRN medium containing calcium and fixed for immunofluorescence at the time points indicated.

### Lentiviral transduction and live imaging of active RhoA.

For live cell imaging of active Rho, colonoids harvested from *Crb3^ERΔIEC^* and *Crb3^fl/fl^* mice were cultured on cell culture imaging dishes with polymer coverslip bottoms (ibidi, 80136) coated with human collagen type IV (33 μg/mL, Sigma-Aldrich, 5533) and rh-Laminin (Thermo Fisher Scientific, A29248). Colonoids were infected with lentivirus packaged with vector pLV-dimericTomato-2xrGBD (Addgene, 176098) using 5–10 μg/mL of polybrene (MilliporeSigma, TR-1003). Forty-eight hours after infection, colonoids were incubated with 200 nM Membright 488 dye (MilliporeSigma, SCT083) for 15 minutes prior to imaging. High-resolution live cell images of active RhoA and plasma membranes were captured using a Zeiss LCM 980 Airyscan 2 equipped with a heated stage insert in the Microscopy and Image Analysis Laboratory Core at the University of Michigan. *Z*-stacks of 0.5 μm thickness for a total of 5–8 μm were acquired to account for differences in the cell height and to acquire all cell-cell junctions in the field of view.

### Image analysis for active Rho and junctional proteins.

Active RhoA intensity in the colonoids derived from *Crb3^ERΔIEC^* and *Crb3^fl/fl^* mice were measured using ImageJ FIJI software. Using plasma membrane signal as a marker of cell-cell junctions, 25-pixel-wide lines were drawn from vertex to vertex to cover the whole junction, including bicellular and tricellular junctions. A similar line (25 pixels wide) was drawn in the cytoplasmic region close to each of the junctions to account for the cell-to-cell variable background signal. Relative fluorescence intensity of active Rho at the cell-cell junction was calculated by deducting the background signal from the junctional signal of dTomato-2xrGBD for respective junctions. A similar quantification method was adopted to measure relative fluorescence intensity of ZO-1 at the cell-cell junctions.

### Line scan of cell-cell junctions.

Intensity and junctional localization of F-actin and ZO-1 were measured using ImageJ FIJI software. Using ZO-1 signal as a marker of cell-cell junctions, a 20-pixel-wide line was drawn across a cell to span across 2 cell-cell junctions and the cytoplasm of the cell. Relative fluorescence intensity at the cell-cell junction was calculated by deducting the background signal from the junctional signal of the respective junctional protein. Background signal was calculated by averaging the intensity of the first and last 5 pixels of the line that spans the junctions.

### Statistics.

Averaged values are expressed as mean ± standard derivation (SD) or mean ± standard error of the mean (SEM). Statistical analysis was performed by 2-tailed Student’s *t* test, 1-way ANOVA, or 2-way ANOVA using GraphPad Prism software. A *P* value of less than 0.05 was considered significant. No samples were excluded from the analysis.

### Study approval.

All animal experiments were approved and performed in accordance with guidelines set by the University Committee on Use and Care of Animals at the University of Michigan. Deidentified human intestinal tissue samples were obtained from the University of Michigan Inflammatory Bowel Diseases biobank.

### Data availability.

All data supporting the findings in this manuscript are available within the paper and its supplement files, including the Supporting Data Values file, and from the corresponding authors upon request.

## Author contributions

S Fan and SV contributed equally to this work. S Fan and SV designed and performed experiments, analyzed data, and drafted the manuscript. VGH, S Flemming, and ARS performed experiments, contributed to data acquisition, and assisted with data interpretation. BM provided critical reagents and contributed to experimental design discussions. CAP and AN conceived and supervised the study, interpreted data, and edited the manuscript. All authors reviewed and approved the final version of the manuscript.

## Funding support

NIH, grant R01 DK129214 (to AN and CAP).

NIH, grant DK59888 (to AN).

## Supplementary Material

Supplemental data

Unedited blot and gel images

Supporting data values

## Version 1 10/22/2025

Electronic publication
