# Supplementary material for: CRB3 and NF2 orchestrate cytoskeletal dynamics to control epithelial barrier assembly
Source: JCI Insight. 2025 Oct 22;10(20):e196350. doi: 10.1172/jci.insight.196350 (PMC12581665; doi:10.1172/jci.insight.196350)
Supplement: Supplemental data [file jciinsight-10-196350-s105.pdf]

## Supplemental Information

# *CRB3 and NF2 orchestrate cytoskeletal dynamics to control epithelial barrier assembly*

**Shuling Fan[1^], Saranyaraajan Varadarajan[1^], Vicky Garcia-Hernandez[1], Sven Flemming[1], Arturo Raya-Sandino[1], Ben Margolis[2], Charles A. Parkos[1*], Asma Nusrat[1*]**

[1]Department of Pathology, University of Michigan Medical School, Ann Arbor, MI 48109, USA.
[2]Department of Internal Medicine, University of Michigan Medical School, Ann Arbor, MI 48109, USA.

^ SF and SV contributed equally to this work.

*Corresponding authors

Correspondence:

Asma Nusrat
University of Michigan Medical School
Department of Pathology
4057 BSRB, 109 Zina Pitcher Place,
Ann Arbor, MI 48109, USA.
Phone: (734) 764-5712
Email: anusrat@umich.edu

Charles A Parkos
University of Michigan Medical School
Department of Pathology
NCRC, Building 35, Rm 30-1537
2800 Plymouth Road,
Ann Arbor, MI 48109, USA.
Phone: (734) 763-6384
Email: cparkos@med.umich.edu

# Supplemental figures

Supplemental figure 1

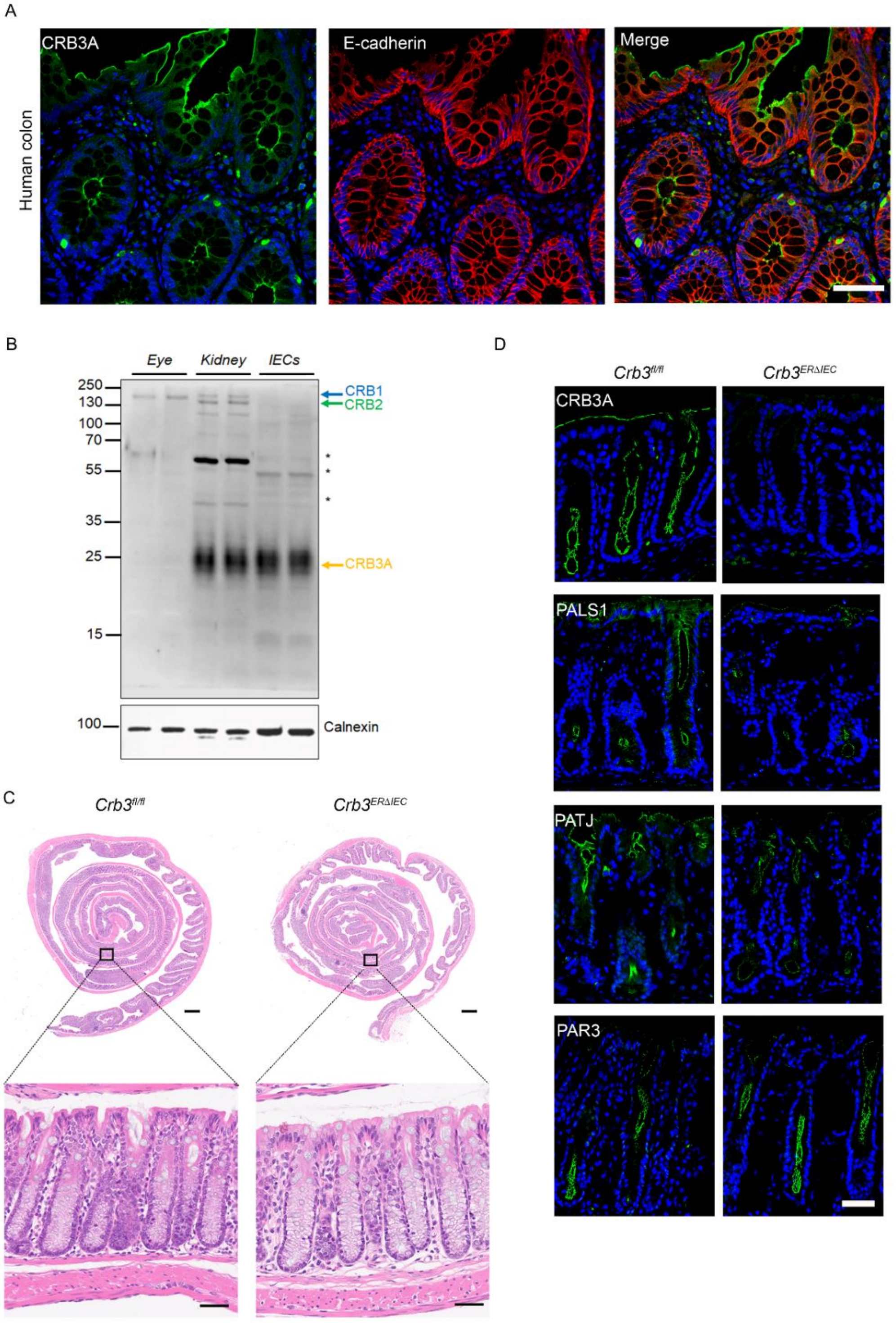

**Supplemental Figure 1: Expression of CRB3 binding partners and histology of colonic mucosa. (a)** Colonic tissue sections of human healthy donor were immunostained for CRB3A (green), E-cadherin (red), and nuclei/ (DAPI, blue). Scale bar = 50 µm; n=2 biological replicates; **(b)** Immunoblotting of CRB3A and Calnexin (loading control) in tissue from murine eye, kidney and IECs. Blue arrow indicates CRB1, green arrow indicates CRB2, and red arrow indicates CRB3A. Asterisks indicate non-specific bands; n=1 experiment, 2 biological replicates; **(c)** Hematoxylin and eosin (H&E) stained Swiss roll colonic tissue sections of tamoxifen-treated *Crb3*$^{fl/fl}$ and *Crb3*$^{ER\Delta IEC}$ mice shows comparable tissue architecture; Scale bar = 500 µm. Black box highlights an area that is enlarged in the bottom panel. Scale bar = 50 µm; n=2 biological replicates; **(d)** Immunofluorescence labeling of colonic tissue sections of tamoxifen-treated *Crb3*$^{fl/fl}$ and *Crb3*$^{ER\Delta IEC}$ mice showing CRB3A, PALS1, PATJ, PAR3, and nuclei (DAPI, blue). Scale bar = 50 µm. n=3 independent experiments; 2 technical replicates each.

Supplemental figure 2

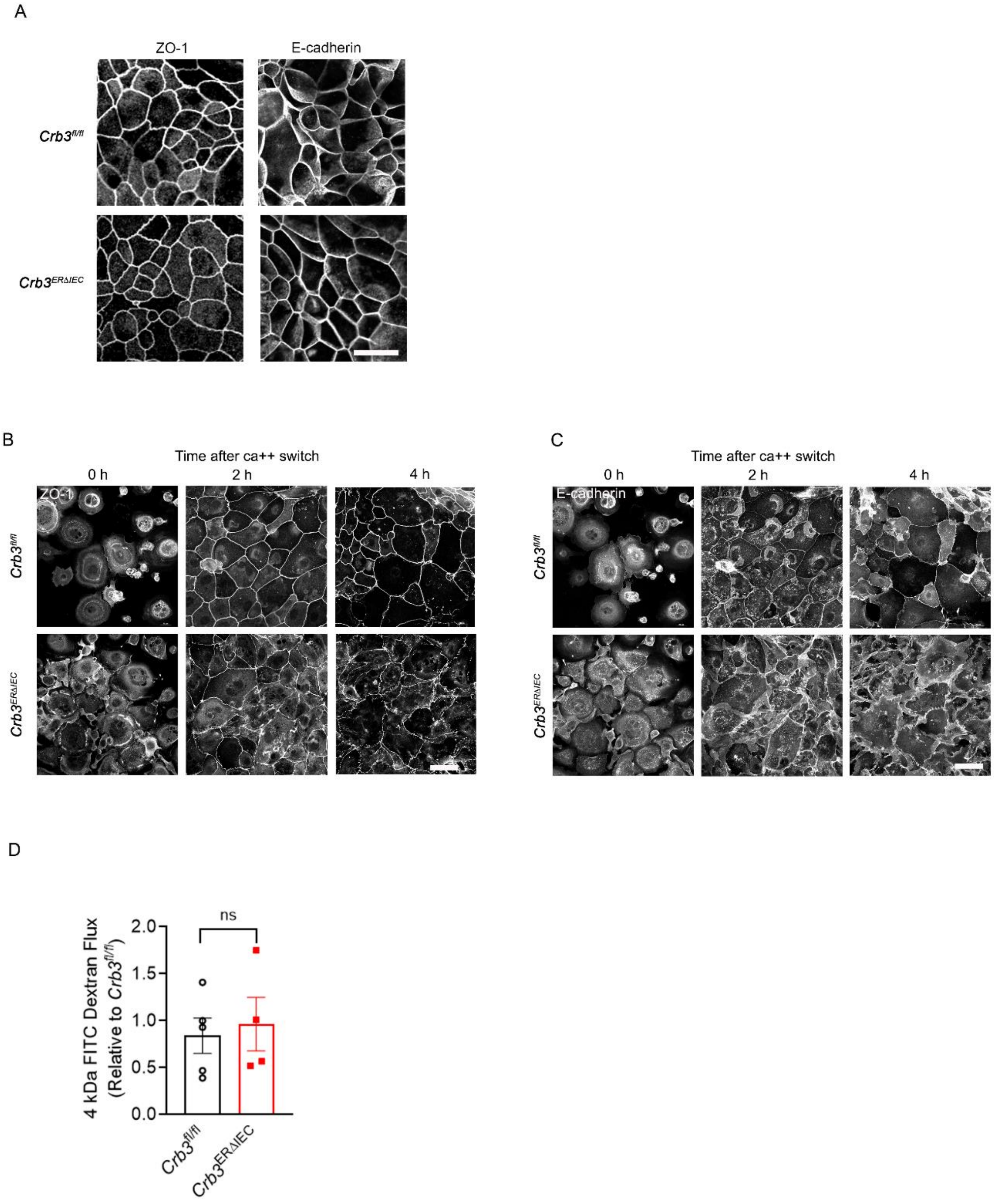


**Supplemental Figure 2: CRB3 is not critical for barrier function and AJC morphology in stable epithelial cell-cell junctions. (a)** Immunofluorescence labeling and confocal images of apical junctional proteins, ZO-1 and E-cadherin, in confluent and polarized colonoids from $Crb3^{fl/fl}$ and $Crb3^{ER\Delta IEC}$ mice grown on transwells filters. Scale bar = 25 μm; n=3 independent experiments; 2 technical replicates each; **(b-c)** Immunofluorescence labeling and confocal images of apical junctional proteins, ZO-1 (**b**) and E-cadherin (**c**) in confluent and polarized colonoids from $Crb3^{fl/fl}$

and *Crb3*$^{ER\Delta IEC}$ mice at 0, 2 and 4h following calcium switch. Scale bar = 25 μm; n=2 independent experiments; **(d)** Intestinal permeability measured by paracellular flux of 4KDa FITC-Dextran in serum in *Crb3*$^{fl/fl}$ and *Crb3*$^{ER\Delta IEC}$ mice at 2 hours post-surgery in mice. Data are mean ± SD. n=1 experiment with five technical replicates. p value calculated using two tailed student's t-test.

Supplemental figure 3

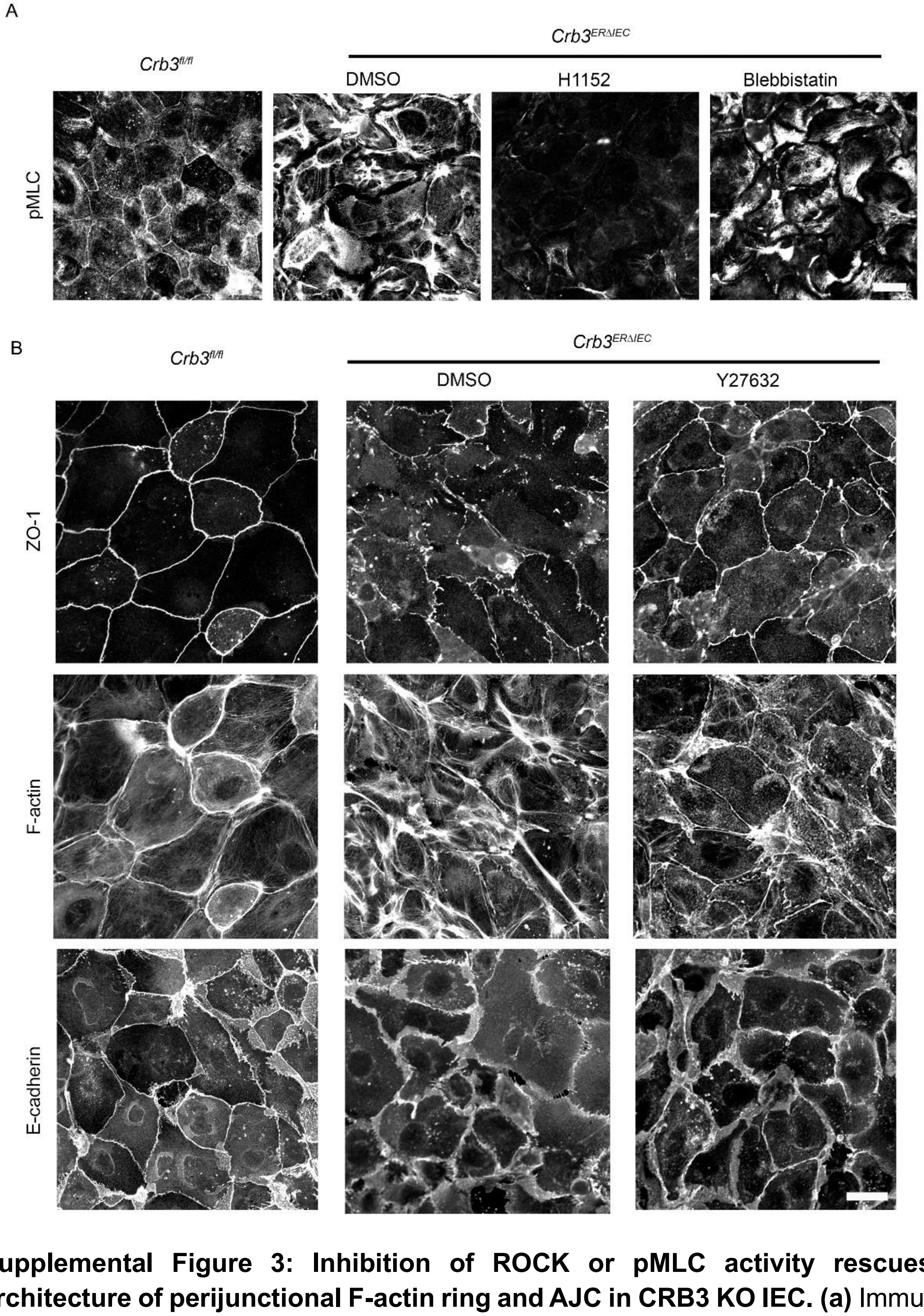


**Supplemental Figure 3: Inhibition of ROCK or pMLC activity rescues the general architecture of perijunctional F-actin ring and AJC in CRB3 KO IEC. (a)** Immunofluorescence labeling of colonoids derived from *Crb3*$^{ER\Delta IEC}$ mice treated with vehicle (DMSO), ROCKII inhibitor (H1152, 10 μM), and Non-Muscle Myosin II ATPase Inhibitor (Blebbistatin, 50 μM) showing p-MLC$^{T18/S19}$ staining; Scale bar = 25 μM. n=3 independent experiments; 2 technical replicates each;

**(b)** Representative confocal images of perijunctional F-actin (Phalloidin), and AJC proteins (ZO-1 and E-cadherin) in *Crb3*$^{fl/fl}$ colonoids and *Crb3*$^{ER\Delta IEC}$ colonoids treated with ROCK II inhibitor (Y27632, 50 µM) or vehicle (DMSO, 0.1%) for 30 minutes. Scale bar = 25 µM. n=3 independent experiments; 2 technical replicates each.

Supplemental Figure 4

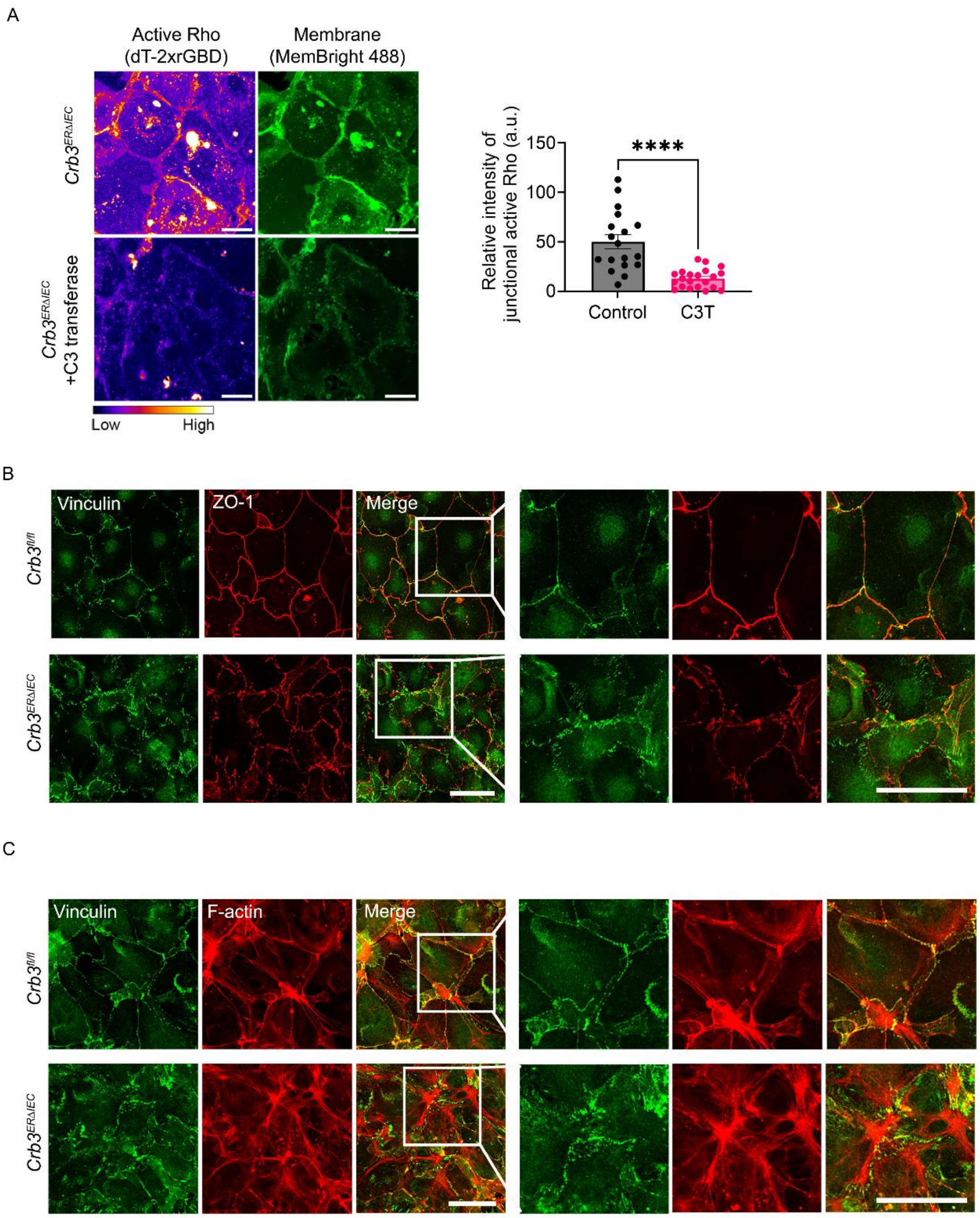


**Supplemental Figure 4: (a)** Representative image of sub-confluent *Crb3*$^{ER\Delta IEC}$ colonoids expressing pLenti-dTomato-2xrGBD (pseudo colored as FIRE LUT) and stained with MemBright 488 plasma membrane dye (green), treated acutely with vehicle (water) and active Rho inhibitor (C3 transferase, 2μg/ml). Images shown are sum of Z-projections. Scale bar = 20 μm; **(Right)** Graph showing the relative intensity of active RhoA at cell-cell junctions normalized to the

background signal of every junction. Graphs are mean ± SEM. Each dot represents an independent junction from 2 independent experiments **** p <0.0001 by Mann-Whitney test. n=18 junctions for control ($Crb3^{ER\Delta IEC}$); n=21 junctions for C3 transferase (C3T+*Crb3*$^{ER\Delta IEC}$); **(b-c)** Representative confocal images of subconfluent colonoids derived from *Crb3*$^{fl/fl}$ and *Crb3*$^{ER\Delta IEC}$ mice immunostained for **(b)** Vinculin (rabbit anti-Vinculin, green) and ZO-1 (red); **(c)** Vinculin (mouse anti-Vinculin, green) and F-actin (red). White box represents zoomed in region shown on the right. n=3 independent experiments; 2 technical replicates each.

Supplemental Figure 5

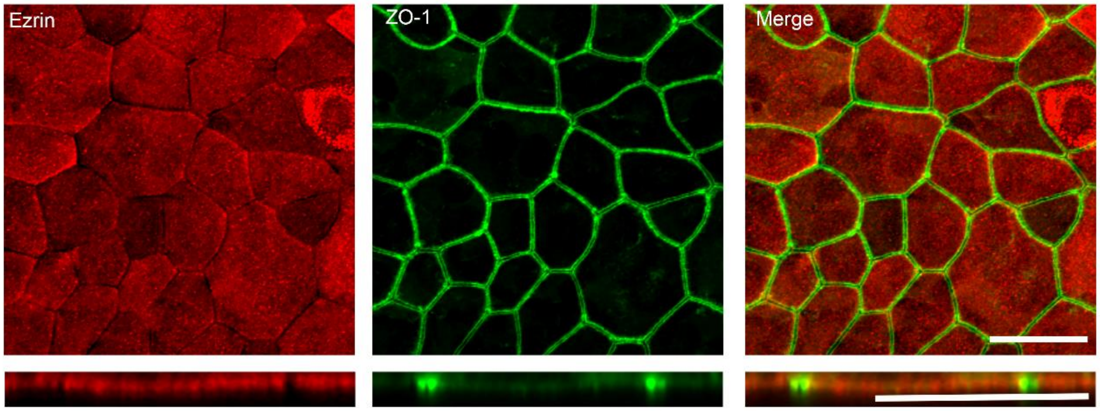


**Supplemental Figure 5: Ezrin localizes to apical membrane.** Immunofluorescence labeling of Ezrin (red) and ZO-1 (green) in confluent primary mouse colonoids grown on permeable support. Bottom panel: Z-projection shows that Ezrin and ZO-1 do not co-localize at TJ. Scale bar = 25 μm. n=2 independent experiments; 2 technical replicates each.

Supplemental figure 6

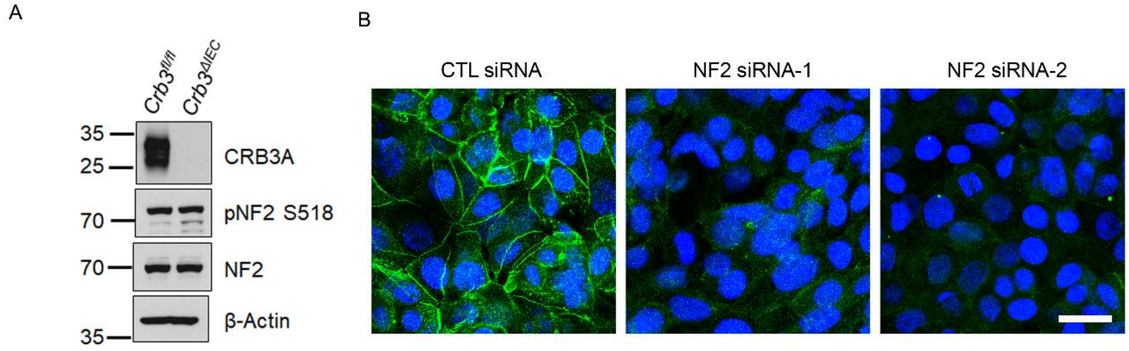


**Supplemental Figure 6: (a) NF2 expression is independent of CRB3.** Immunoblotting of CRB3A, NF2, pNF2S518 and β-actin (loading control) in colonoids derived from $Crb3^{fl/fl}$ and $Crb3^{ER\Delta IEC}$ mice. n=3 independent experiments; **(b) Efficiency of NF2 knockdown in model IECs.** Immunofluorescence labeling of SK CO-15 IECs transfected with control (scramble) or two independent NF2 siRNA showing NF2 (green) and DAPI (nuclei, blue). Scale bar = 25 μm. n=3 independent experiments.

Supplemental figure 7

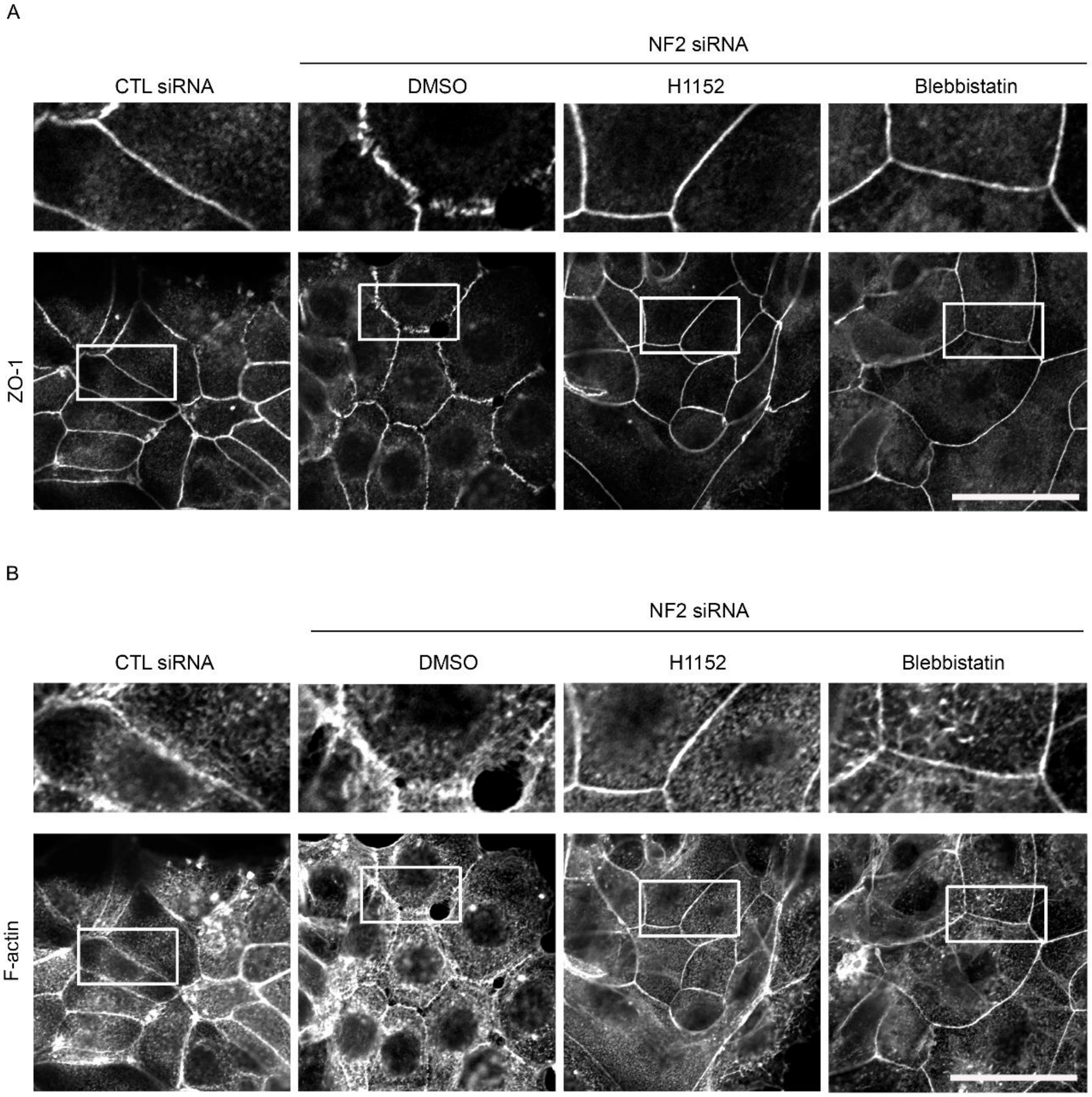


**Supplemental Figure 7: Inhibition of ROCK or pMLC activity rescues the general architecture of perijunctional F-actin ring and AJC in NF2 KD IEC. (a-b)** Representative confocal images of TJ proteins ZO-1 **(a)** and perijunctional F-actin (Phalloidin, **b**) in SK CO-15 treated with NF2 siRNA or CTL siRNA for 72 hours, and treated with ROCK II inhibitor (H1152, 10 µM), Myosin II Inhibitor (Blebbistain, 50 µM) or vehicle (DMSO, 0.1%) for 30 minutes. White box shows the region enlarged in the panel above. Scale bar = 25 µm. n=3 independent experiments; 2 technical replicates each.

**Supplemental table 1**

| Specificity | species | Suppliers | Catalog number | Dilution |
|---|---|---|---|---|
| CRB3a | rabbit | In-house | N/A | WB 1:4000; IF 1:500 |
| β-actin | mouse | Sigma Aldrich | A5441 | WB 1:5000 |
| Merlin | rabbit | Cell Signaling Technology | 12888 | IF 1:100 |
| Merlin | rabbit | Cell Signaling Technology | 6995 | WB 1:1000; IP 1:100 |
| p-Merlin S518 | rabbit | Cell Signaling Technology | 9163 | IP 1:100 |
| Myc-tag | mouse | Cell Signaling Technology | 2276 | IP 1:100 |
| Myc-tag | rabbit | Cell Signaling Technology | 2278 | WB 1:2000 |
| PALS1 | rabbit | In-house | N/A | WB 1:1000; IF 1:200 |
| PARD3(PAR3) | rabbit | Novus | NBP1-88861 | WB 1:1000; IF 1:100 |
| PATJ | rabbit | In-house | N/A | IF 1:100 |
| PATJ/INADL | rabbit | LifeSpan Bioscience | LS-C410011 | WB 1:1000 |
| p-MLCT18/S19 | rabbit | Cell Signaling Technology | 3674 | IF 1:250 |
| p-MLC S19 | rabbit | Cell Signaling Technology | 3671 | IF 1:250 |
| ZO-1 | mouse | ThermoFisher | 33-9100 | WB 1:1000; IF 1:250 |
| ZO-1 | rabbit | ThermoFisher | 40-2200 | IF 1:250 |
| E-cadherin | goat | R & D System | AF648 | WB 1:2000; IF 1:500 |
| Ezrin | mouse | ThermoFisher | 35-7300 | WB 1:1000 |
| β-catenin | mouse | BD Bioscience | 610153 | WB 1:1000; IF 1:500 |
| Alexa Fluor™ 488 Phalloidin | | ThermoFisher | A12379 | IF 1:400 |
| Alexa Fluor™ 555 Phalloidin | | ThermoFisher | A34055 | IF 1:400 |
| Vinculin | mouse | ThermoFisher | 14-9777-82 | IF 1:100 |
| Vinculin | rabbit | ThermoFisher | 700062 | IF 1:100 |
| Calnexin | rabbit | Abcam | ab22595 | WB 1:4000 |
