# Supplementary material for: CRB3 and NF2 orchestrate cytoskeletal dynamics to control epithelial barrier assembly
Source: JCI Insight. 2025 Oct 22;10(20):e196350. doi: 10.1172/jci.insight.196350 (PMC12581665; doi:10.1172/jci.insight.196350)
Supplement: Unedited blot and gel images [file jciinsight-10-196350-s106.pdf]

Full unedited blot for Figure 1c

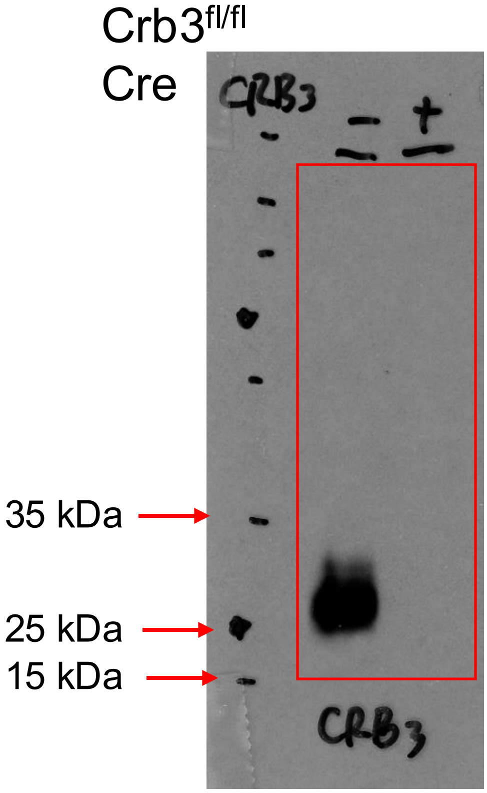

CRB3a blot

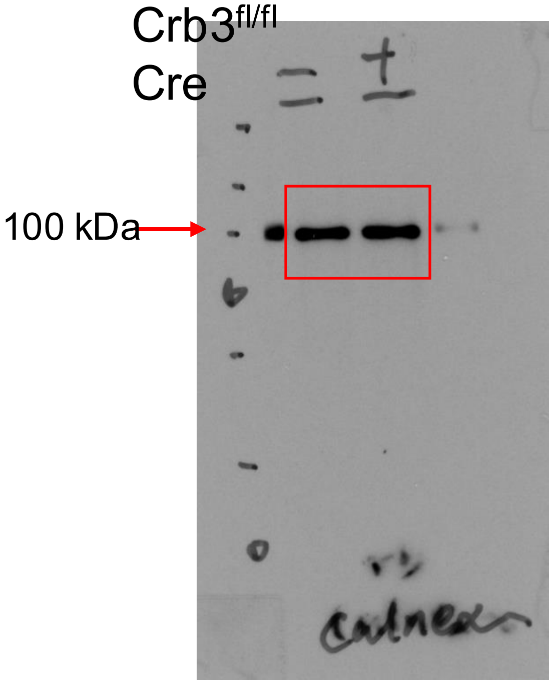

Calnexin blot

Full unedited blot for Figure 2b

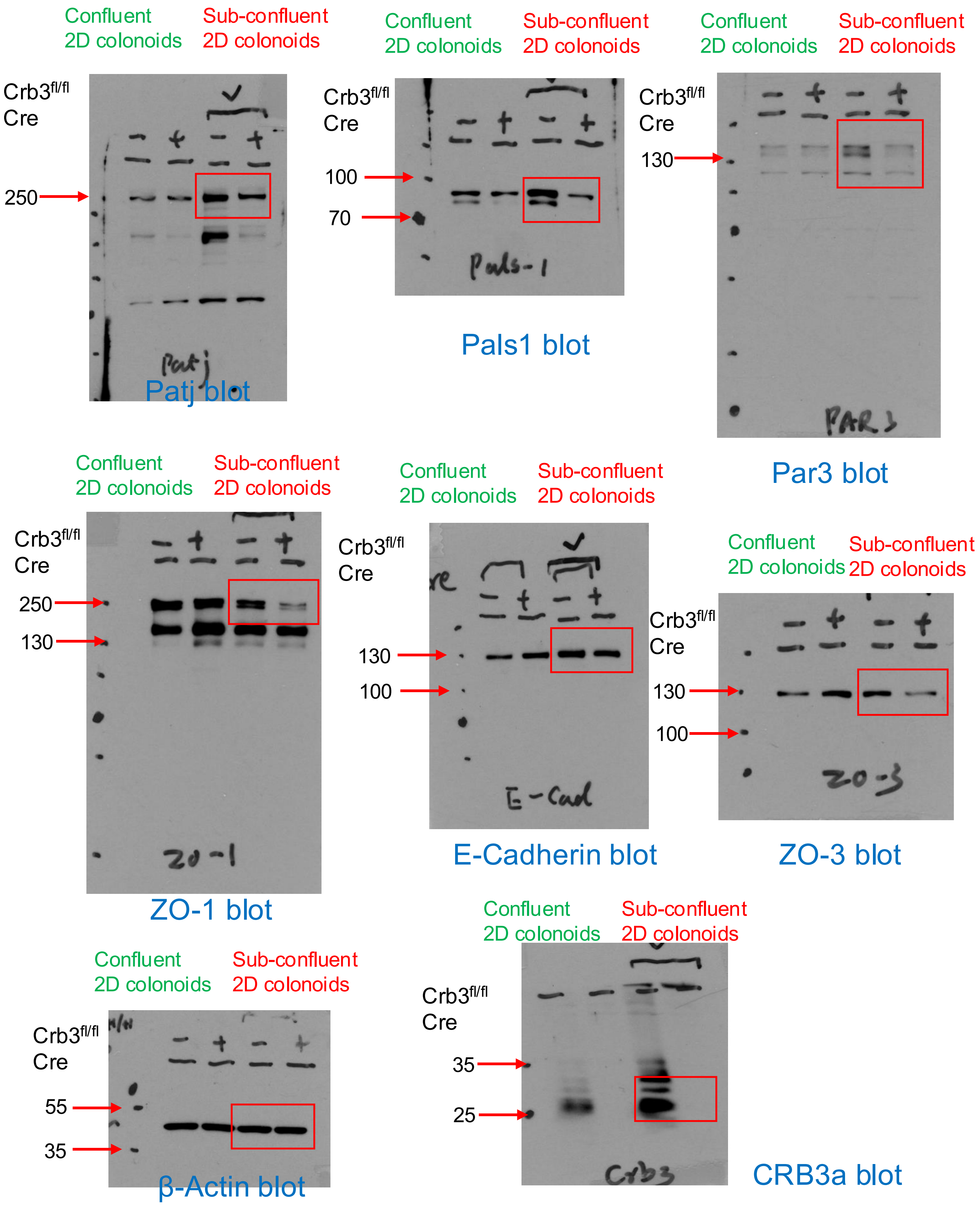

Full unedited blot for Figure 7b

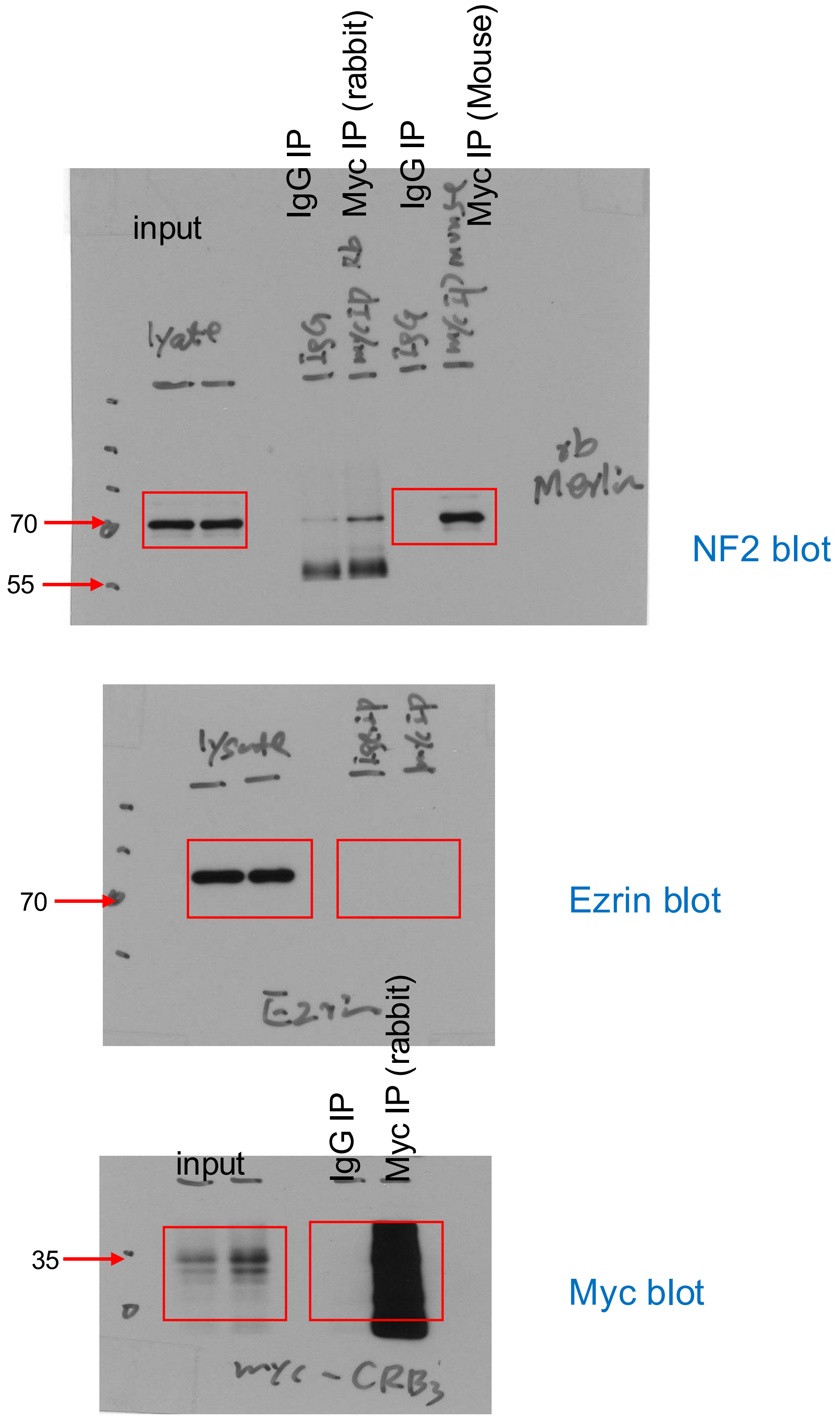

Full unedited blot for Figure 7c

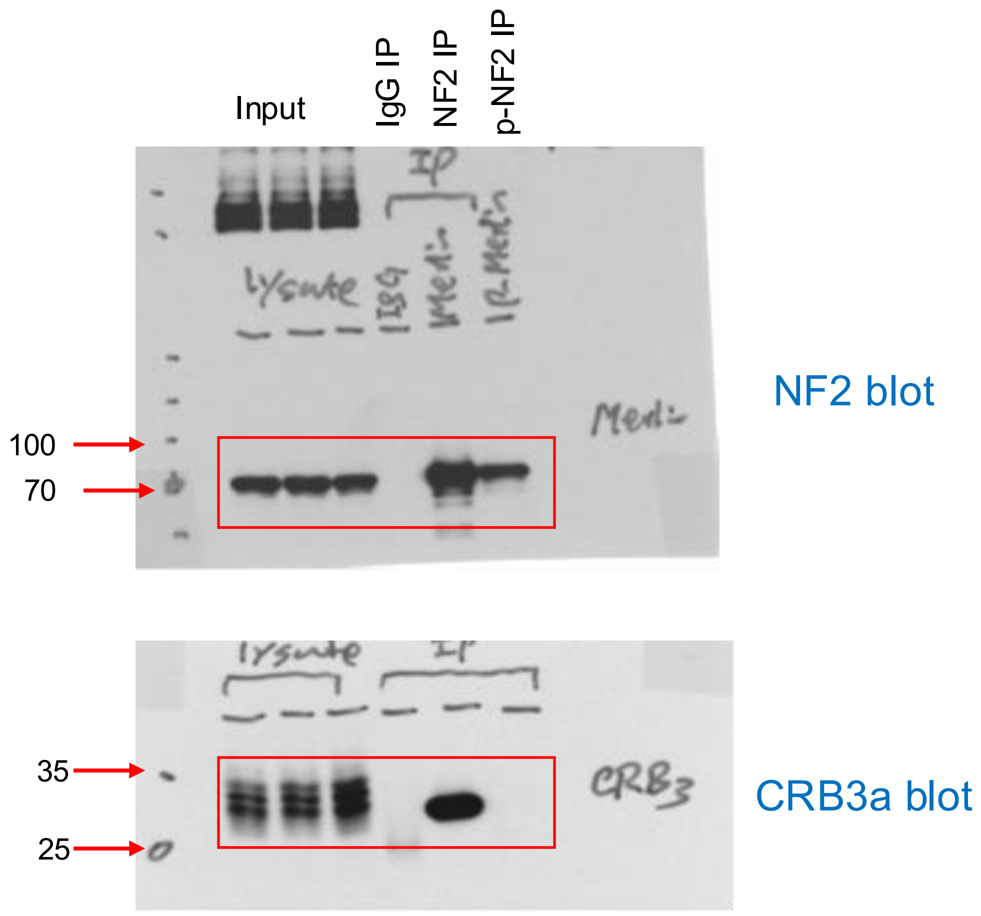

Full unedited blot for Figure 7e

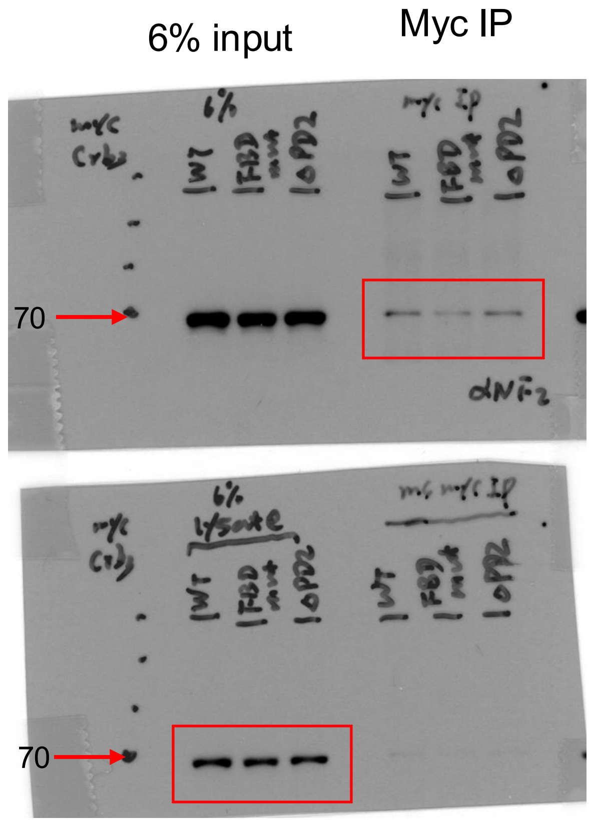

NF2 blot

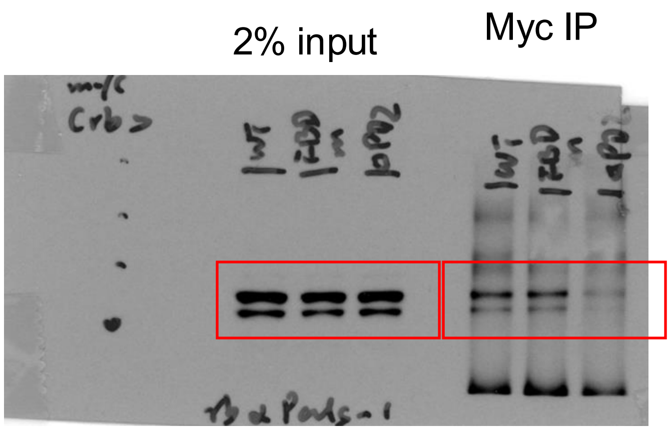

PALS1 blot

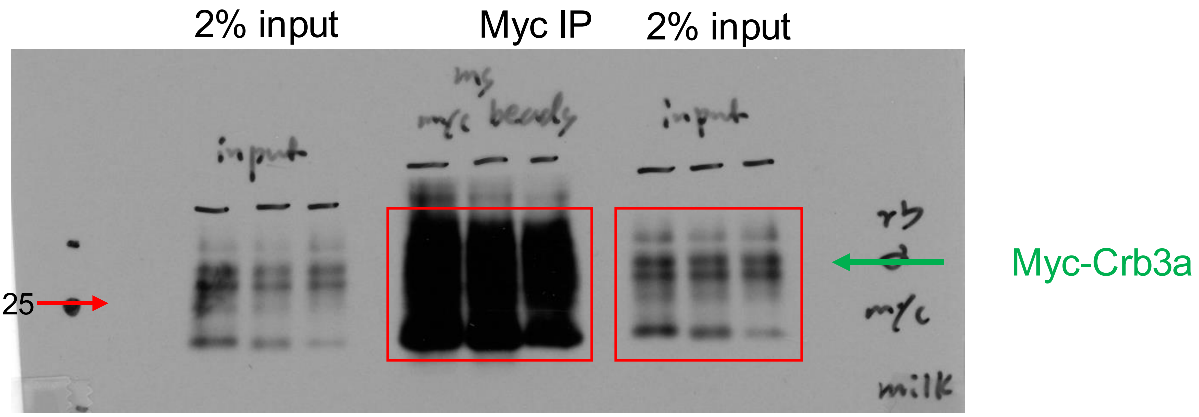

Myc blot

Full unedited blot for Figure 8

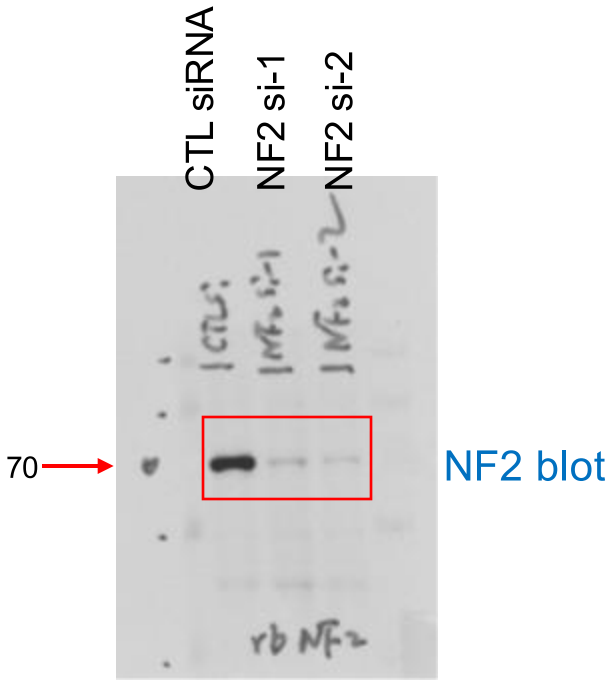

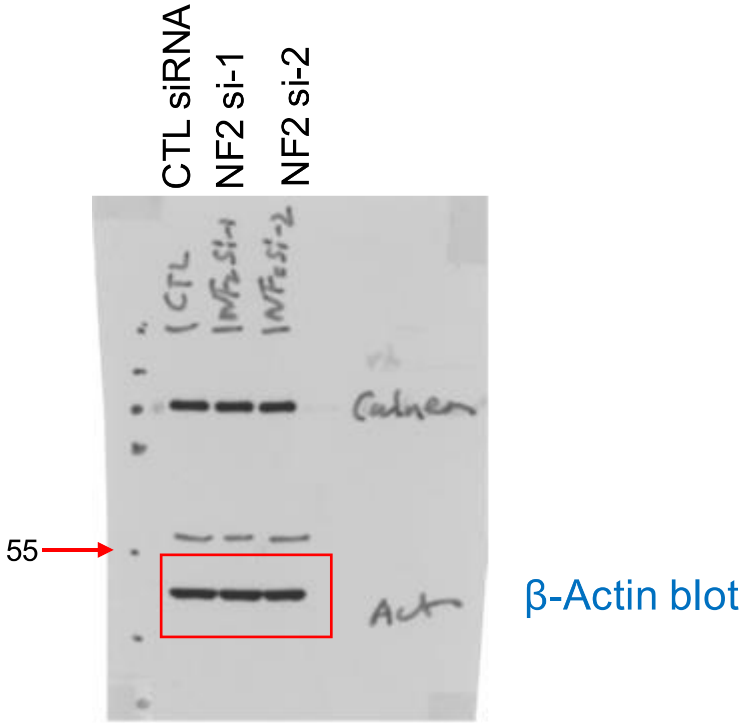

Full unedited blot for Supplemental Figure 1b

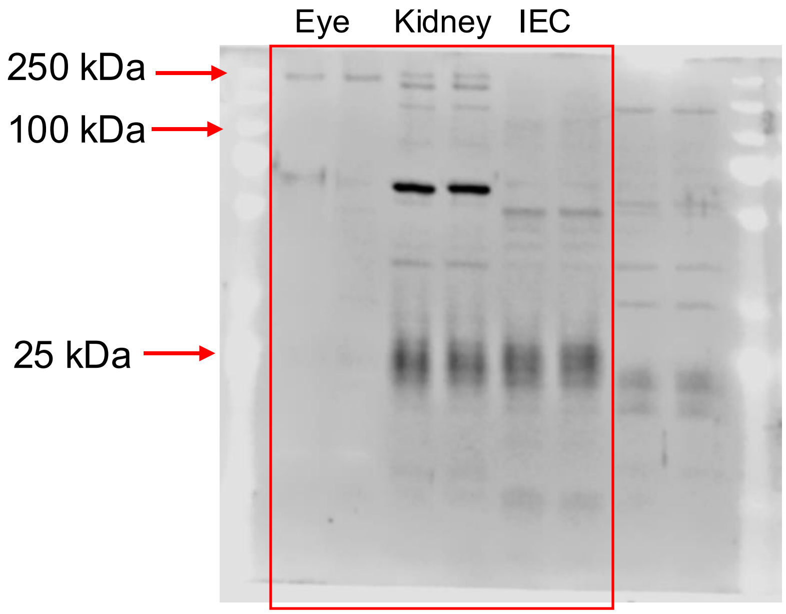

Crb3a blot

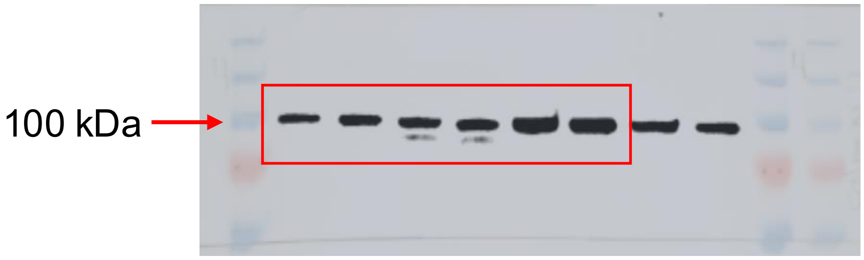

Calnexin blot

Full unedited blot for Supplemental Figure 6a

Crb3$^{fl/fl}$
Cre

25 kDa

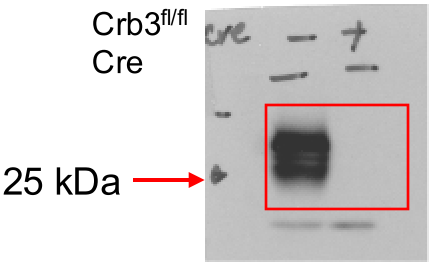

Crb3a blot

Crb3$^{fl/fl}$
Cre

70 kDa

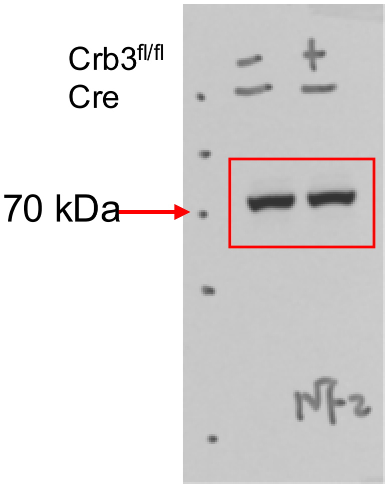

NF2 blot

Crb3$^{fl/fl}$
Cre

70 kDa

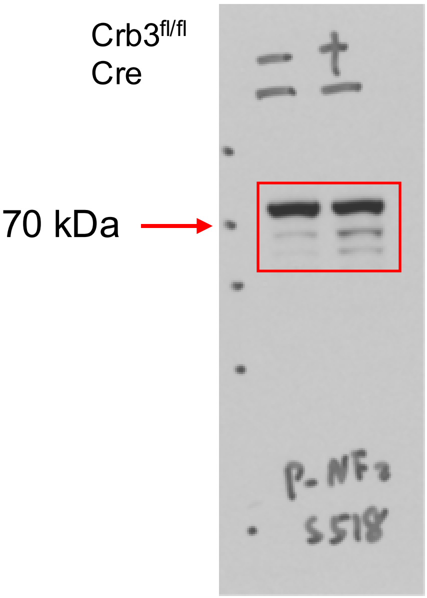

p-NF2 S518 blot

Crb3$^{fl/fl}$
Cre

25 kDa

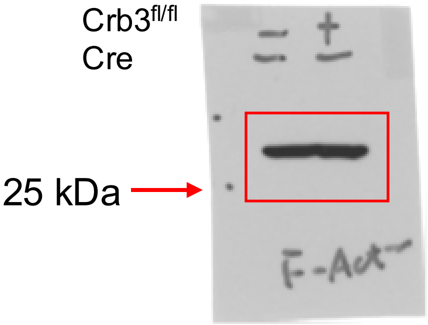

β-Actin blot
